# Cost of Nine Pediatric Infectious Illnesses in Low- and Middle-Income Countries: A Systematic Review of Cost-of-Illness Studies

**DOI:** 10.1007/s40273-020-00940-4

**Published:** 2020-08-04

**Authors:** Gatien de Broucker, So Yoon Sim, Logan Brenzel, Margaret Gross, Bryan Patenaude, Dagna O. Constenla

**Affiliations:** 1 International Vaccine Access Center, Johns Hopkins Bloomberg School of Public Health, Baltimore, MD USA; 2 Welch Medical Library, Johns Hopkins Medical Institutions, Baltimore, MD USA; 3 Bill and Melinda Gates Foundation, Seattle, WA USA; 4 Department of International Health, Johns Hopkins Bloomberg School of Public Health, Baltimore, MD USA; 5 GlaxoSmithKline Plc, Panama City, Panama; 6 International Vaccine Access Center, 415 North Washington Street, Suite #530, Baltimore, MD 21231 USA

## Abstract

**Background:**

Cost-of-illness data from empirical studies provide insights into the use of healthcare resources including both expenditures and the opportunity cost related to receiving treatment.

**Objective:**

The objective of this systematic review was to gather cost data and relevant parameters for hepatitis B, pneumonia, meningitis, encephalitis caused by Japanese encephalitis, rubella, yellow fever, measles, influenza, and acute gastroenteritis in children in low- and middle-income countries.

**Data Sources:**

Peer-reviewed studies published in public health, medical, and economic journals indexed in PubMed (MEDLINE), Embase, and EconLit.

**Study Eligibility Criteria, Participants, and Interventions:**

Studies must (1) be peer reviewed, (2) be published in 2000–2016, (3) provide cost data for one of the nine diseases in children aged under 5 years in low- and middle-income countries, and (4) generated from primary data collection.

**Limitations:**

We cannot exclude missing a few articles in our review. Measures were taken to reduce this risk. Several articles published since 2016 are omitted from the systematic review results, these articles are included in the discussion.

**Conclusions and Implications of Key Findings:**

The review yielded 37 articles and 267 sets of cost estimates. We found no cost-of-illness studies with cost estimates for hepatitis B, measles, rubella, or yellow fever from primary data. Most estimates were from countries in Gavi preparatory (28%) and accelerated (28%) transition, followed by those who are initiating self-financing (22%) and those not eligible for Gavi support (19%). Thirteen articles compared household expenses to manage illnesses with income and two articles with other household expenses, such as food, clothing, and rent. An episode of illness represented 1–75% of the household’s monthly income or 10–83% of its monthly expenses. Articles that presented both household and government perspectives showed that most often governments incurred greater costs than households, including non-medical and indirect costs, across countries of all income statuses, with a few notable exceptions. Although limited for low- and middle-income country settings, cost estimates generated from primary data collection provided a ‘real-world’ estimate of the economic burden of vaccine-preventable diseases. Additional information on whether common situations preventing the application of official clinical guidelines (such as medication stock-outs) occurred would help reveal deficiencies in the health system. Improving the availability of cost-of-illness evidence can inform the public policy agenda about healthcare priorities and can help to operationalize the healthcare budget in local health systems to respond adequately to the burden of illness in the community.

**Electronic supplementary material:**

The online version of this article (10.1007/s40273-020-00940-4) contains supplementary material, which is available to authorized users.

## Key Points for Decision Makers

**Table:** 

Few studies with primary data collection were conducted to assess the cost of vaccine-preventable diseases in low- and middle-income countries: there were none for measles, hepatitis B, rubella, or yellow fever.
Cost estimates generated from primary data collection can provide a ‘real-world’ estimate of the economic burden of vaccine-preventable diseases. Additional information on whether common situations that may have influenced the application of official clinical guidelines (such as medication stock-outs) occurred, would help reveal deficiencies in the health system.
Private healthcare is underrepresented. Estimating costs for private facility use offers a useful comparison with government-funded healthcare and provide insights for engaging private stakeholders in the universal health coverage strategy.

## Introduction

Vaccines are considered a highly cost-effective, public health intervention that can reduce the healthcare and household costs incurred by vaccine-preventable illnesses (VPD). To measure the scale of the economic burden from VPD, we rely on cost-of-illness (COI) studies to assess the costs associated with a specified illness and perspective [1, 2]. Cost-of-illness studies estimate the costs associated with treating and managing illnesses paid for by patients, governments, insurers, and charitable organizations. They also reveal costs borne by households to obtain healthcare, from travel and accommodations costs to the loss of income. As such, COI studies reflect a comprehensive view of the economic burden of disease and uncovers gaps in the health system that compromise equal access to healthcare.

In low- and middle-income countries (LMIC), defined by the World Bank lending groups [3], where governments rely on loans and external funding to finance public healthcare and immunization programs, COI studies are insightful to identify diseases that consume the most resources and aggravates inequalities in the population. Cost-of-illness studies relying on primary data collection, as opposed to modeling and secondary sources, would be best suited to capture all types of costs from different perspectives, using interviews or surveys of patients, caregivers, or healthcare professionals as well as medical records and administrative data.

Yet, prior reviews show that the number of studies focusing on diseases in LMIC with primary data collection paled in contrast to studies in high-income countries [4, 5]. For instance, of the 365 articles (1996–2006) reported in Akobundu et al. [5] only 20 focused on LMIC (and most using modeled data). We aim to bridge this apparent gap and review the costs associated with selected VPD in children in LMIC: hepatitis B, pneumonia, influenza, meningitis, encephalitis caused by the Japanese encephalitis virus (JE), rubella, yellow fever, measles, and acute gastroenteritis.

## Methods

### Literature Review

This systematic review followed the PRISMA (Preferred Reporting Items for Systematic Reviews and Meta-Analyses) statement guidelines [6] and focused on studies that generated COI estimates from primary data collection, excluding those that generated COI estimates through modeling or secondary data sources. Studies that focused on infectious diseases that can potentially be prevented by vaccines including hepatitis B, pneumonia, meningitis, influenza, encephalitis caused by JE, rubella, yellow fever (YF), measles, and acute gastroenteritis (GE) in children aged under 5 years in LMICs were considered. For GE, we considered articles that presented data on GE either without specified etiology (e.g., diarrhea) or caused by rotavirus. We conducted an online literature search using three electronic databases: PubMed, Embase, and EconLit. We used a combination of controlled vocabulary and keyword terms that included the following concepts: (1) hepatitis B, pneumonia, influenza, meningitis, JE, rubella, YF, measles or GE, (2) cost data, (3) children, and (4) low- and middle-income countries (see Appendix 3 in electronic supplementary material (ESM)). We originally ran the search query for all infectious diseases reported on the World Health Organization’s (WHO’s) list before choosing to include only articles relevant to one of the nine diseases. We generated a list of keywords for LMIC based on the World Bank country classifications. We limited the search to peer-reviewed articles within the date range 2000–2016.

While we searched in English only, we included articles in French, Spanish, and Portuguese. All titles and abstracts relevant to our study were retrieved and searched for full text. Records from all databases were imported on 6 March, 2017. Potentially relevant articles published since then (as of 13 May 2020) are cited in the discussion. In addition to stand-alone COI studies, we included cost–benefit, cost-effectiveness, or cost-utility analyses if they produced COI estimates from primary data collection generated by the authors and not based on secondary sources.

### Screening Process

Each article was examined by two reviewers over the four phases of the review process. Phase I examined the eligibility of each article by reviewing its title and abstract. For articles to be considered for inclusion, they had to: (1) be peer-reviewed articles published between 2000 and 2016; (2) provide cost data on VPDs in children aged under 5 years in LMICs; and (3) collect primary data. Phase II assessed eligibility based on the full text.

**Fig. 1:**
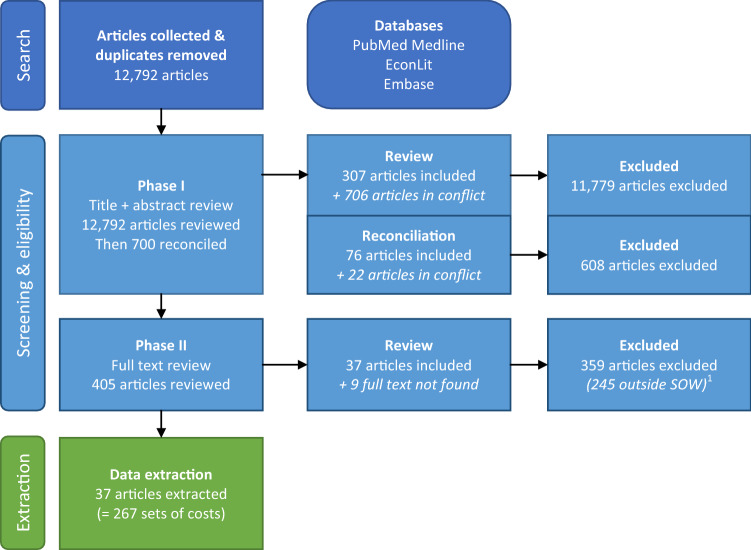
Preferred Reporting Items for Systematic Reviews and Meta-Analyses (PRISMA) flowchart. ^1^We considered as outside our scope of work (SOW) the 245 articles that did not present any cost estimate for at least one of the eight diseases of interest

Instead of focusing on all childhood infectious illnesses, we narrowed our focus to nine VPDs: hepatitis B, all-cause pneumonia, all-cause influenza, all-cause meningitis, encephalitis caused by JE, rubella, YF, measles, and GE. This update to the screening process is reflected in the numbers of articles excluded in phase II (see Fig. 1).

Articles with conflicting reviewer decisions on eligibility were reviewed by the same two reviewers through a second round (labeled as “reconciliation”). The original response was removed so as not to make the reviewer aware of the decision made by the other reviewer.

### Data Extraction

An article can produce more than one set of cost estimates, each set being associated with a specific perspective or scenario. For an article that reported cost estimates for the household and the healthcare system perspectives, comparing data from two different countries, and three VPDs (e.g., non-severe pneumonia, severe pneumonia, and meningitis), the authors may have presented 12 different sets of COI estimates: one per combination of parameters (two perspectives × two countries × three VPDs).

To highlight the multiple COI estimates reported per article, we disaggregated the articles into “sets”, effectively differentiating COI estimates as the authors report them. In this context, a “set” was defined as a *scenario or a combination of parameters producing one distribution of costs, thus one cost estimate*. Consequently, if there was more than one distribution of costs in an article, more than one set of costs was reported (Appendix 1 in ESM).

Data extraction was performed by two reviewers. Each set of costs extracted was checked by the second reviewer. Variances in data extraction between the two reviewers, such as the identification of sets of costs and the classification of the reported costs, were thoroughly discussed until agreement was reached. Variables and descriptions in the dataset were edited to reflect this.

### Presentation of Cost Data

“Empirical COI evidence” was defined as the set of costs associated with an episode of the illness, estimated based on primary data collection [1, 2, 4, 5, 7]. To examine how cost estimates were structured and aggregated, we can refer to the Global Health Costing Consortium’s guidelines with the “intervention unit” cost associated with the cost per person diagnosed with the illness [8]. The selection of costs included when costing an illness is defined by an epidemiological approach (i.e., incidence or prevalence based) rather than as programmatic cost centered around the delivery of a specific intervention. It implies that a COI estimate can include costs outside the scope of an intervention (e.g., care provided at home, long-term productivity losses).

Costs are reported as service costs [8], except for the household perspective for which costs are classified as either direct or indirect costs [2]. Service costs include all costs associated with supplies such as medications for any services provided at the facility, and they integrate the healthcare facility operating costs and capital costs. For the household perspective, direct costs include the out-of-pocket payments incurred to access health services. Direct costs include medical (e.g., hospital charges, medications) and non-medical expenses (e.g., transportation to and from healthcare facilities, meals, and lodging for the caregivers). Indirect costs include the economic or opportunity costs incurred to receive or provide care including the costs of reduced productivity or lost time from paid employment resulting from illness or treatment [2]. A comprehensive list of costs is available in Appendix 4 in ESM.

The baseline cost (mean and/or median), sample size (*n*), and estimates of the error margin such as the confidence interval, interquartile values, and/or range are all presented [9]. The approach used to collect the cost data, either prospective or retrospective, is also presented. A summary of the cost estimates by perspective is presented in Tables 2, 3, and 4. All costs were converted in 2018 US$ using the average foreign exchange rates for the year [10] and the country’s consumer price index derived from the International Monetary Fund [11]. Furthermore, we discuss how the selected articles compare in light of the recommendations of prior reviews of COI studies, more particularly Clabaugh and Ward [4].

## Results

The search yielded a total of 12,792 unique articles after duplicates were removed (Fig. 1). After reconciliation, 405 articles moved on from the first phase of screening. Moving on to the full-text review (phase II), we narrowed the diseases of interest to nine diseases: articles focusing on other diseases were still included in the review. Reviewers examined the full text of 396 articles (nine articles did not have the full text available, they were likely poster abstracts) and found that 114 articles did not present costs generated empirically and 245 articles did not present data on any of the nine diseases of interest. We made a final selection of 37 articles. The level of inter-reviewer agreement (Cohen’s kappa) was 68.1% (substantial agreement) by the end of the screening process [12].

### Countries, Illness, and Populations

From the 37 articles, we drew 267 different sets of costs. Five of the nine VPDs were identified from the 267 sets of costs: GE (116 sets, 43%), pneumonia (121, 45%), meningitis (22 sets, 8%), JE (three sets, 1%), influenza (two sets, 1%), and other illnesses (three sets, 1%). In the latter, two sets only specified “other” and “very severe disease” related to pneumococcal infection and one specified acute otitis media. We found no estimate for hepatitis B, measles, rubella, and YF in children. Children between the age of 1 and 59 months were the main age group under study (218 sets, 81%), followed by children aged 0–3 years (35 sets, 13%). Children aged older than 5 years were also included in six sets of costs, 2% (Table 1).

**Table 1:** Summary of articles

Reference (first author, year)	Diseases and type of care	Case confirmation	Country and year of costing	Age group	Perspective, sector, level of care	Study design	Types of costs			Costs itemized?	Funder
							Direct medical	Direct non-medical	Indirect		
Jacob (2016) [16]	Gastroenteritis, IP, OP	Clinical assessment only	India, N/S	0–5 years	HH, private, tertiary	Prospective cross-sectional study using WHO (2005) guideline as costing methodology	Y	Y	N	N	GO
Bar-Zeev (2016) [14]	Gastroenteritis, IP, OP	Laboratory tests	Malawi, 2014	0–5 years	HH, HC, public primary, tertiary	CEA with a prospective cross-sectional COI study using WHO (2005) guideline as costing methodology	Y	N	N	N	NGO
Usuf (2016) [39]	Pneumonia, pneumococcal sepsis, bacterial meningitis, IP, OP	Laboratory tests	Gambia, N/S	0–5 years	HH, HC, S, public, primary, tertiary	Prospective and retrospective cross-sectional study	Y	Y	Y	Y	NGO
Ngabo (2016) [17]	Gastroenteritis, IP	Laboratory tests	Rwanda, 2014	0–5 years	HH, HC, TP, public, secondary, tertiary	Prospective cross-sectional study using WHO (2005) guideline as costing methodology	Y	Y	Y	Y	NGO
Patel (2015) [40]	Pneumonia, IP, OP	Radiology	India, N/S	3–59 months	HH, public and private, secondary	RCT with a prospective cross-sectional COI study	Y	Y	Y	N	PR, NGO
Loganathan (2015) [32]	Gastroenteritis (rotavirus and non-rotavirus), IP	Laboratory tests	Malaysia, 2013	0–5 years	HH, public, tertiary	Prospective cross-sectional study	Y	Y	Y	N	GO, PR
Soltani (2015) [67]	Gastroenteritis, IP	Laboratory tests	Tunisia, 2009	0–5 years	HC, public secondary, tertiary	Retrospective cross-sectional study	Y	N	N	N	NGO
Alkoshi (2015) [38]	Gastroenteritis, IP	Laboratory tests	Libyan Arab Jamahiriya, N/S	0–5 years	HH, HC, public, tertiary	Prospective and retrospective cross-sectional study	Y	Y	Y	N	GO
Burke (2014) [18]	Gastroenteritis, IP, OP	Clinical assessment only	Bolivia, N/S	0–5 years	HH, public and private, tertiary	Prospective cross-sectional study using WHO (2005) guideline as costing methodology	Y	Y	Y	N	GO, NGO
Le (2014) [37]	Pneumonia, meningitis, UD	Laboratory tests	Vietnam, 2012	0–5 years	HH, HC, S, public, tertiary	Prospective and retrospective cross-sectional study using WHO (2005) guideline as costing methodology	Y	Y	Y	Y	NGO
Zhou (2013) [68]	Influenza, IP	Laboratory tests	China, 2010	0–15 years	HC, public, tertiary	Prospective and retrospective cross-sectional study	Y	N	N	Y	GO
Burke (2013) [19]	Gastroenteritis, IP, OP	Clinical assessment only	Bolivia, N/S	0–5 years	HH, public and private, tertiary	Prospective cross-sectional study using WHO (2005) guideline as costing methodology	Y	Y	Y	N	GO, NGO
Sinha (2012) [26]	Acute lower respiratory tract infections, IP	Laboratory tests	South Africa, 2010	0–5 years	HH, HC, public, tertiary	Prospective and retrospective cross-sectional study	Y	Y	Y	Y	PR, NGO
Temple (2012) [27]	Pneumonia, OP	Clinical assessment only	Fiji, N/S	0–5 years	HH, HC, S, public, primary, tertiary	Prospective cross-sectional study	Y	Y	Y	Y	N/S
Anh (2010) [25]	Pneumonia, meningitis, IP	Radiology and laboratory tests	Vietnam, 2006	0–5 years	HC, public, tertiary	Prospective cross-sectional study using MOF Vietnam guidelines as costing methodology	Y	N	N	Y	GO, NGO
MacIntyre (2010) [24]	Gastroenteritis (rotavirus and non-rotavirus), IP	Laboratory tests	South Africa, N/S	0–5 years	HH, HC, public, tertiary	Prospective and retrospective cross-sectional study using WHO (unpublished) as costing methodology	Y	Y	Y	N	NGO
Chai (2009) [30]	Gastroenteritis (rotavirus and non-rotavirus), IP	Laboratory tests	Malaysia, 2007	0–12 years	HH, public, tertiary	Prospective cross-sectional study	Y	Y	Y	N	PR
Tate (2009) [20]	Gastroenteritis (rotavirus), IP, OP	Clinical assessment only	India, 2009	0–5 years	HH, private, secondary	Prospective cross-sectional study using WHO (2005) guideline as costing methodology	Y	Y	Y	N	GO
Flem (2009) [21]	Gastroenteritis (rotavirus), IP	Clinical assessment only	Kyrgyzstan, 2008	0–5 years	HH, HC, S, public and private, secondary, tertiary	CEA with a prospective cross-sectional COI study using WHO (2005) guideline as costing methodology	Y	Y	Y	N	GO, NGO
Tate (2009) [33]	Gastroenteritis (rotavirus), IP, OP	Clinical assessment only	Kenya, 2007	0–5 years	HH, HC, public, primary, tertiary	Prospective and retrospective cross-sectional study	Y	Y	Y	N	GO, NGO
Chola (2009) [23]	Pneumonia, gastroenteritis, IP, OP	Clinical assessment only	Zambia, 2006	0–5 years	HC, public, secondary	Retrospective cross-sectional study using WHO (1994) cost analysis guideline as costing methodology	Y	N	N	N	GO
Lee (2007) [28]	Gastroenteritis (rotavirus), IP	Laboratory tests	Malaysia, 2002	0–14 years	HC, public, tertiary	Retrospective cross-sectional study	Y	N	N	Y	N/S
Constenla (2007) [69]^a^	Pneumonia, pneumococcal pneumonia, meningitis, acute otitis media, IP, OP	Radiology	Brazil, 2004	0–5 years	HC, public, primary, secondary, tertiary	Prospective and retrospective cross-sectional study	Y	N	N	N	NGO
Platonov (2006) [31]	Hib meningitis, IP	Laboratory tests	Russian Federation, 2001	0–5 years	S, public, tertiary	Retrospective cross-sectional study	Y	N	Y	N	NGO
Fischer (2005) [36]	Gastroenteritis, IP, OP	Clinical assessment only	Vietnam, 2004	0–5 years	S, public, secondary,	CEA with a prospective cross-sectional COI study	Y	Y	Y	N	GO, NGO
Guzmán (2005) [48]	Pneumonia, pneumococcal pneumonia, IP	Radiology	Colombia, 2002	0–2 years	S, public and private, tertiary	Prospective cross-sectional study	Y	N	Y	Y	GO, PR
Riewpaiboon (2016) [70]	Gastroenteritis (rotavirus and non-rotavirus), UD	Laboratory tests	Vietnam, 2014	0–5 years	S, public and private, primary, secondary, tertiary	Prospective cross-sectional study	Y	Y	Y	N	GO, PR, NGO
Mathew (2016) [44]	Gastroenteritis (rotavirus), IP	Laboratory tests	India, N/S	0–5 years	HC, private, tertiary	Retrospective cross-sectional study	Y	N	N	N	GO
Griffiths (2013) [15]	Japanese encephalitis, IP	Laboratory tests	Nepal, 2011	1 month to 14 years	HH, public, tertiary	Prospective cross-sectional study	Y	Y	Y	N	NGO
Wilopo (2009) [22]	Gastroenteritis (rotavirus), IP, OP	Clinical assessment only	Indonesia, 2007	0–5 years	S, public and private, primary, secondary	CEA with a prospective cross-sectional COI study using WHO (2005) guideline as costing methodology	Y	Y	Y	N	GO, NGO
Hussain (2006) [29]	Pneumonia, meningitis, sepsis, IP, OP	Laboratory tests	Pakistan, 2001	0–5 years	HC, HC, public and private, primary, secondary, tertiary	Retrospective cross-sectional study focused on activity-based costing	Y	N	N	N	PR
Sadruddin (2012) [71]	Pneumonia, IP, OP	Radiology and laboratory tests	Pakistan, N/S	0–5 years	HH, public and private, primary	Prospective cross-sectional study	Y	Y	Y	N	GO, NGO
Ashraf (2010) [42]	Pneumonia, IP, OP	Clinical assessment only	Bangladesh, N/S	0–5 years	S, public, primary, secondary,	RCT with a prospective cross-sectional COI study	Y	N	N	N	NGO
Madsen (2009) [34]	Pneumonia, IP	Radiology and laboratory tests	India, 2008	0–3 years	HH, HC, private, secondary, tertiary	Prospective cross-sectional study	Y	Y	Y	Y	N/S
Ayieko (2009) [72]	Pneumonia, meningitis, IP	Radiology and laboratory tests	Kenya, 2005	0–5 years	HC, HC, public and private primary, secondary, tertiary	Prospective cross-sectional study	Y	N	N	Y	NGO
Hussain (2008) [35]	Pneumonia, IP, OP	Radiology and laboratory tests	Pakistan, 2002	0–3 years	HH, HC, S, public and private, primary, secondary, tertiary	Prospective and retrospective cross-sectional study	Y	Y	Y	N	N/S
Kitchin (2011) [45]	Pneumonia, IP	Radiology and laboratory tests	South Africa, 2007	0–5 years	HC, public, tertiary	Retrospective cross-sectional study	Y	N	N	N	PR

*CEA* cost-effectiveness analysis, *COI* cost-of-illness, *G* government perspective, *GO* public organization, *HC* healthcare perspective, *HH* household perspective, *IP* inpatient care, *MOF* ministry of finance, *N* not available, *NGO* multi-lateral agency or non-governmental organization, *N/S* not specified, *OP* outpatient care, *PR* private for-profit organization, *S* societal perspective, *RCT* randomized controlled trial, *TP* third-party payer perspective, *UD* undistinguished inpatient/outpatient care, *Y* available, *WHO* World Health Organization

^a^Constenla (2007) also featured costs for Chile and Uruguay, two high-income countries. Only costs for Brazil, an upper-middle-income country, were included in the analysis

The largest portion of these costs came from countries in Sub-Saharan Africa (97 sets, 36%), followed by South Asia (80 sets, 30%), East Asia and Pacific (67, 25%), Latin America and the Caribbean (16 sets, 6%), Europe and Central Asia (four sets, 2%), and the Middle East and North Africa (three sets, 1%). Based on Gavi’s 2018 Annual Progress Report [13], most sets came from countries in preparatory (75 sets, 28%) and accelerated transitions (also 75 sets, 28%), followed by countries initiating self-financing (59 sets, 22%) and non-Gavi-eligible countries (51 sets, 19%). Indonesia was the only fully self-financing country with a COI study (seven sets, 3%).

Most COI evidence was generated in countries where publicly funded healthcare was available. The most commonly reported perspectives were the government perspective where we found 96 sets of costs (36%) and the household perspective (79 sets, 30%) for which most of the data collection was performed in public healthcare facilities. Studies that adopted the healthcare and the household perspectives also adopted a societal perspective in 47 sets of costs (18%). Thirty sets of costs (11%) were reported from the perspective of private healthcare providers without further details on whether the costs were transferred to users or other sources of revenue. Eleven sets of costs (4%) took a healthcare provider perspective, not differentiating what was paid by the government and by the private sector. One article reported costs borne by health insurance, hence taking a third-party payer perspective in four sets of the costs (2%).

Most sets of costs were associated with COI in urban settings (127 sets, 48%). Eighty-three sets (31%) were associated with rural settings and 57 sets with mixed urban and rural settings (21%). In our selected articles, each set of costs could combine costs for more than one facility level: 164 sets of costs (61%) included costs from tertiary healthcare facilities, 81 (30%) from secondary healthcare facilities, and 93 (35%) from primary healthcare facilities.

### Scope and Methods

Of the 37 articles, 11 (30%) integrated a COI component with primary data collection within a larger study. Five of 37 papers were cost-effectiveness analyses, four were burden of disease studies, and two were randomized controlled trials. The remaining 26 articles were stand-alone COI studies. These COI studies took an incidence-based cross-sectional approach, defining the COI around the healthcare facility visit for an acute episode of the illness. While they focused on the acute episode of the illness, most sets of costs adopting the household perspective included a follow-up period, 7–14 days after the initial episode.

To identify VPD cases, 23 articles reported costs with laboratory-confirmed cases (GE, meningitis, JE) and nine with radiology-confirmed cases (pneumonia). Among these, six studies used both laboratory testing and radiology. Eleven articles relied on clinical assessment alone to identify cases.

Twenty-one articles (57%) took a prospective approach to data collection, seven (19%) took a retrospective approach, and nine (24%) combined prospective and retrospective approaches. Costs from the household perspective were always estimated prospectively as these relied on caregiver responses. Ten articles (27%) included caregiver interviews. Most of them (eight articles, 22%) performed a follow-up interview 7–14 days after the initial interview. One article performed interviews 6 weeks later [14], another 5–12 months afterward [15].

Among the articles assessing the cost of GE (19 articles), half of them used an established costing method. Eight used the WHO guidelines to estimate the economic burden of diarrhea published in 2005 [14, 16–22], one used the WHO guideline for cost analysis in primary healthcare published in 1994 [23], and one used unpublished WHO guidelines cited as “WHO (U Griffiths, R Rheingans, D Walker, unpublished data)” [24]. One article focusing on pneumonia and meningitis used the national guideline from the Ministry of Finance (Vietnam) to estimate capital costs [25]. None reported a qualitative component (expert consultations) to identify potential costs for households and the healthcare system.

### Types of Costs

All 37 articles presented direct medical costs. Twenty-three (62%) also presented direct non-medical costs and 24 (65%) presented indirect costs. Twenty-two articles presented all three types of costs. Direct medical costs included medications and medical procedures, and most articles presented such medical costs aggregated.

Five articles (14%) included overhead costs [26–29] and two (5%) added discounted capital costs [25, 29]. Four articles [25–27, 29] counted them as part of the medical costs, while one [28] included them as part of an “indirect cost” from the provider perspective.

Direct non-medical costs included the cost of transportation (21 of 37 articles), meals during the facility visit (9 of 37), caregiver accommodations (3 of 37), and other costs related to childcare (8 of 37) such as diapers, visitors’ gifts, and transportation for non-caregivers. Four articles presented aggregated direct non-medical costs only and one article aggregated all direct medical and non-medical costs as a “total cost of admission”.

Indirect costs were based on income loss for the caregiver(s) (18 of 37 articles) and time loss for the caregiver because of disability (1 of 37 articles). The other four articles presented only an aggregated indirect cost value and did not describe cost composition. All indirect costs were estimated through a human capital approach, considering the productivity loss of caregivers. Among the articles reporting income loss, six articles estimated income loss for the surveyed caregiver only [15, 17, 24, 26, 30, 31], eight estimated it for both the father and the mother of the sick child [18, 19, 22, 32–36], and three assessed income loss for all reported caregivers [21, 27, 37]. One article did not disclose whose income was lost [38].

Most articles reported the costs during the year of data collection or the year following data collection, without any correction for inflation. One article [26] used unpublished costs originally collected in 1998–2000 and adjusted them to the 2010 national currency value. Several articles [18, 19, 24, 38–40] collected costs over several years and did not specify the year of the currency value; we assumed the year of the currency was the starting year for data collection to correct for the missing base year.

### Cost Estimates

Direct medical, non-medical, and indirect costs per episode for inpatient care were greater than for outpatient care for the household perspective (Table 2). There was no apparent trend between these costs and country income status. For pneumonia and GE episodes, household direct medical costs had a range of $3.52–$125.39 per hospitalized case and $0–$53.87 per ambulatory case. For JE, the medical costs ranged between $577.65 and $1268.84. Non-medical costs were similar across country income statuses and diseases, $1.21–$28.29 (inpatient), and $0–$8.94 (outpatient), with an exception for meningitis where they were much higher: $28.78–179.46 (mixed inpatient/outpatient). Ranges of reported indirect costs were similar between diseases and overlapped across country income statuses with larger variations with higher income statuses: $11.21–$41.31 (low income), $2.25–$90.78 (lower-middle income), and $0.55–$214.55 (upper-middle income) (Tables 2, 3, and 4).

**Table 2:** Household cost estimates by article (2018 US$). Mean and median estimates provided by different sets of costs within an article are reported as a range with the differences in estimates explained in the description

Source (author, year)	Mean (or median^c^) estimates of household costs				Description of difference in estimates
	Direct medical	Direct non-medical	Indirect	Overall	
**Gastroenteritis**					
Bolivia, lower-middle-income country in Gavi accelerated transition					
Hospitalized					
Burke (2014) [18]	$16.96	$17.96	$36.91	$90.78	
Burke (2013) [19]^c^	$27.30–$18.68	$8.04–$12.65	$23.94–$40.58	$64.45–$82.88	Urban vs rural settings
Ambulatory					
Burke (2014) [18]	$53.87	$10.97	$25.94	$49.88	
Burke (2013) [19]^c^	$0.00	$5.75–$8.94	$2.45–$17.24	$21.43–$35.18	Urban vs rural settings
Not distinguished					
Burke (2014) [18]^c^	$10.97–$73.82	$12.97–$18.96	$24.94–$52.87	$39.91–$117.72	With vs without national health insurance; public vs private
India, upper-middle-income country in Gavi accelerated transition					
Hospitalized					
Jacob (2016) [16]^c^	$125.39	$10.54	–	–	
Tate (2009) [20]^c^	$117.80	$1.26	–	–	
Ambulatory					
Jacob (2016) [16]^c^	$10.21	$3.28	–	–	
Tate (2009) [20]^c^	$3.11–$3.48	$0.58–$0.67	–	–	Ambulatory care vs emergency room
Kenya, lower-middle-income country in Gavi preparatory transition					
Hospitalized					
Tate (2009) [33]	^b^	$2.70	$11.33	–	
Ambulatory					
Tate (2009) [33]	^b^	$0.00	$2.58	–	
Kyrgyzstan, lower-middle-income country in Gavi preparatory transition					
Hospitalized					
Flem (2009) [21]	^b^	^b^	$2.25	$43.06	
Libyan Arab Jamahiriya, upper-middle-income country and not Gavi-eligible					
Hospitalized					
Alkoshi (2015) [38]	–	^b^	$40.89	$184.20	
Malawi, low-income country and in Gavi initial self-financing					
Hospitalized					
Bar-Zeev (2016) [14]	$9.54–$15.17	–	–	–	Severe vs non-severe case; urban tertiary hospital vs rural health center
Ambulatory					
Bar-Zeev (2016) [14]	$0.52–7.27	–	–	–	Severe vs non-severe case; urban tertiary hospital vs rural health center
Not distinguished					
Bar-Zeev (2016) [14]	$1.93–$15.17	–	–	–	Severe vs non-severe case; urban tertiary hospital vs rural health center
Malaysia, upper-middle-income country and not Gavi-eligible					
Hospitalized					
Loganathan (2015) [32]	$3.52–$90.61	$3.52–$22.87	$19.35–$94.13	$28.15–$198.82	Rotavirus vs non-rotavirus; urban vs rural settings
Chai (2009) [30]	^b^	^b^	$61.21–$81.50	$171.98–$214.55	Rotavirus vs non-rotavirus
Rwanda, low-income country and in Gavi initial self-financing					
Hospitalized					
Ngabo (2016) [17]	$6.83–$10.41	^b^	$11.21–$28.2	–	Urban vs rural settings; tertiary vs secondary hospitals
South Africa, upper-middle-income country and not Gavi-eligible					
Hospitalized					
MacIntyre (2010) [24]	^b^	^b^	$0.55	$15.54	
**Pneumonia**					
Fiji, upper-middle-income country and not Gavi-eligible					
Ambulatory					
Temple (2012) [27]	$0.08–$0.59	$3.23–$8.34	$0.42–$1.22	$4.48–$11.19	Tertiary hospital vs primary healthcare clinic
Gambia, low-income country and in Gavi initial self-financing					
Hospitalized					
Usuf (2016) [39]	^b^	^b^	–	$18.69–$41.31^a^	Urban tertiary hospital vs rural health center
India, upper-middle-income country in Gavi accelerated transition					
Hospitalized					
Patel (2015) [40]^c^	$3.92–$5.01	$1.21–$4.98	$0.00	–	Hospital vs unsupervised (home) amoxicillin treatment
Madsen (2009) [34]	$36.92–$140.79	$10.98–$28.29	$6.41–7.72	$54.30–$146.82	Tertiary vs secondary hospital
Ambulatory					
Patel (2015) [40]^c^	$2.51–$3.14	$1.74–$1.81	$0.00	–	Hospital vs unsupervised (home) amoxicillin treatment
Not distinguished					
Patel (2015) [40]^c^	$6.28–$6.49	$2.72–$6.79	$0.00	$12.05–$18.18	Hospital vs unsupervised (home) amoxicillin treatment
Pakistan, lower-middle-income country in Gavi preparatory transition					
Hospitalized					
Sadruddin (2012) [71]	$7.08	$2.07	$2.08	$11.23	
Hussain (2008) [35]	–	–	–	$19.41–$38.82^a^	Severe vs very severe illness
Ambulatory					
Sadruddin (2012) [71]	$1.92	$0.03	$0.21	$2.15	
Hussain (2008) [35]	–	–	–	$6.45^a^	
Not distinguished					
Hussain (2008) [35]	$8.04	$13.25	$6.35	–	
South Africa, upper-middle-income country and not Gavi-eligible					
Hospitalized					
Sinha (2012) [26]	^b^	$6.17–$14.23	$1.21–$2.34	$10.97–$17.72	HIV + vs HIV-; HIV pediatric ward vs short-stay ward
Vietnam, upper-middle-income country and not Gavi-eligible					
Not distinguished					
Le (2014) [37]	^b^	$58.67	$69.02	$236.98^a^	
**Meningitis**					
Gambia, low-income country and in Gavi initial self-financing					
Not distinguished					
Usuf (2016) [39]	$19.83–$57.01	$28.78–$56.07	–	–	Urban tertiary hospital vs rural health center
Vietnam, upper-middle-income country and not Gavi-eligible					
Not distinguished					
Le (2014) [37]	^b^	$179.46	$161.06	$422.20^a^	
**Japanese encephalitis**					
Nepal, low-income country and in Gavi initial self-financing					
Hospitalized					
Griffiths (2013) [15]^**†**^	$577.65–$1268.84	–	$91.50–$98.11	$492.77–$831.20	Severe vs non-severe illness

*HIV* human immunodeficiency virus

There were no costs from the household perspective for influenza, hepatitis B, measles, yellow fever, or rubella. Country income statuses defined by the World Bank lending groups in 2020 [3]. Gavi status defined by the 2018 Gavi Annual Progress Report [13]

^a^Overall cost includes all direct costs and does not include any indirect cost

^b^Aggregate cost was not provided by the authors and could not be calculated. Itemized costs (e.g., fees, medications, transportation) are available

^c^Article reported median costs instead of mean costs

**Table 3:** Government cost estimates by article (2018 US$). Mean and median estimates provided by different sets of costs within an article are reported as a range with the differences in estimates explained in the description

Source (author, year)	Mean (or median^b^) estimates of government costs				Description of difference in estimates
	Bed/stay	Medications	Tests/procedures	Total	
**Gastroenteritis**					
Kenya, lower-middle-income country in Gavi preparatory transition					
Hospitalized					
Tate (2009) [33]	$169.45	$7.69	$0.86	$178.00	
Ambulatory					
Tate (2009) [33]	N/A	$2.44	$0.43	$11.98	
Libyan Arab Jamahiriya, upper-middle-income country and not Gavi-eligible					
Hospitalized					
Alkoshi (2015) [38]	$337.08	$103.96	$30.07	$471.03	
Malawi, low-income country and in Gavi initial self-financing					
Hospitalized					
Bar-Zeev (2016) [14]	–	–	–	$46.76–$59.59	Severe vs non-severe case; urban tertiary hospital vs rural health center
Ambulatory					
Bar-Zeev (2016) [14]	–	–	–	$7.48–$15.83	Severe vs non-severe case; urban tertiary hospital vs rural health center
Not distinguished					
Bar-Zeev (2016) [14]	–	–	–	$16.52–$49.39	Severe vs non-severe case; urban tertiary hospital vs rural health center
Malaysia, upper-middle-income country and not Gavi-eligible					
Hospitalized					
Lee (2007) [28]	$38.71	$2.15	$40.75	$297.00	
South Africa, upper-middle-income country and not Gavi-eligible					
Hospitalized					
MacIntyre (2010( [24]	$938.67–$1194.47	$6.45–$13.43	*$47.69–$60.38*	$1013.32–$1277.97	Retrospective (2004) vs prospective (2005) data collection
Zambia, lower-middle-income country and not Gavi-eligible					
Hospitalized					
Chola (2009) [23]	–	–	–	$80.02	
Ambulatory					
Chola (2009) [23]	–	–	–	$26.67	
**Pneumonia**					
Brazil, upper-middle-income country and not Gavi-eligible					
Hospitalized					
Constenla (2007) [69]^c^	$494.67	$98.93	$52.07	$645.67	
Not distinguished					
Constenla (2007) [69]^c^	$41.66	$67.69	$13.89	$130.18	
Gambia, low-income country and in Gavi initial self-financing					
Hospitalized					
Usuf (2016) [39]	$20.37–$31.96	$8.41–$15.70	$15.70–$26.35	$56.92–$84.95	Urban tertiary hospital vs rural health center
Ambulatory					
Usuf (2016) [39]	N/A	$2.34–$2.99	$2.80–$2.99	$5.14–$9.16	Urban tertiary hospital vs rural health center
Kenya, lower-middle-income country in Gavi preparatory transition					
Hospitalized					
Ayieko (2009) [72]	$96.78–$346.78	$5.40–$36.72	$8.54–$45.27	$130.86–$428.79	Different facility level; different regions
Pakistan, lower-middle-income country in Gavi preparatory transition					
Hospitalized					
Hussain (2006) [29]	–	$22.54–$75.25	$32.02–$111.90	$132.81–$442.54	Severe vs non-severe illness
Hussain (2008) [35]	–	–	–	$0.91–$500.39	Severe vs very severe illness; different facility level; different healthcare facilities
Ambulatory					
Hussain (2006) [29]	N/A	$0.07–$0.18	$0.16–$0.47	$0.35–$162.54	Severe vs non-severe illness; different facility level
Hussain (2008) [35]	N/A	–	–	$0.33–$124.00	Different facility level; different healthcare facilities
South Africa, upper-middle-income country and not Gavi-eligible					
Hospitalized					
Kitchin (2011) [45]^**†**^	$127.20–$381.61	$0.00	$103.86–$188.86	$253.91–$615.67	HIV + vs HIV-; HIV pediatric ward vs pediatric ICU
Sinha (2012) [26]	–	$6.51–$781.63	^a^	$205.07–$6623.99	HIV + vs HIV-; HIV pediatric ward vs short-stay ward; retrospective (2000) vs prospective (2001) data collection
Vietnam, upper-middle-income country and not Gavi-eligible					
Hospitalized					
Anh (2010) [25]	$18.25–$22.32	$18.16–$86.25	$6.24–16.65	$15.64–$122.71	Different severity levels; probable vs confirmed case
Not distinguished					
Le (2014) [37]	$123.09	$33.36	$28.76	$207.07	
Zambia, lower-middle-income country and not Gavi-eligible					
Hospitalized					
Chola (2009) [23]	–	–	–	$220.57	
Ambulatory					
Chola (2009) [23]	–	–	–	$49.24	
**Meningitis**					
Brazil, upper-middle-income country and not Gavi-eligible					
Hospitalized					
Constenla (2007) [69]^c^	$1249.68	$546.74	$171.83	$1968.25	
Gambia, low-income country and in Gavi initial self-financing					
Not distinguished					
Usuf (2016) [39]	$25.23–$27.10	$14.39–$40.47	$40.47–$79.63	$97.38–$122.43	Urban tertiary hospital vs rural health center
Kenya, lower-middle-income country in Gavi preparatory transition					
Hospitalized					
Ayieko (2009) [72]	$272.83–$540.69	$44.56–$106.58	$32.17–$108.56	$392.14–$538.83	Different facility levels; public vs private
Pakistan, lower-middle-income country in Gavi preparatory transition					
Hospitalized					
Hussain (2006) [29]	–	$705.30	$720.19	$3918.52	
Vietnam, upper-middle-income country and not Gavi-eligible					
Hospitalized					
Anh (2010) [25]	$26.22–$52.27	$35.60–$127.72	$15.68–$53.50	$77.51–$291.39	Meningitis etiology
Not distinguished					
Le (2014) [37]	$140.35	$103.54	$69.02	$345.12	
**Influenza**					
China, upper-middle-income country and not Gavi-eligible					
Hospitalized					
Zhou (2013) [68]	$29.85–$33.59	$208.98–$218.94	$57.22–$46.03	$323.43–$337.11	Healthy children vs children with underlying condition

*HIV* human immunodeficiency virus, *ICU* intensive care unit

There were no costs from the government perspective for Japanese encephalitis, hepatitis B, measles, yellow fever, or rubella. Country income statuses defined by the World Bank lending groups in 2020 [3]. Gavi status defined by the 2018 Gavi Annual Progress Report [13]

^a^Aggregate cost was not provided by the authors and could not be calculated. Disaggregated costs are available

^b^Article reported median costs instead of mean costs

^c^Constenla (2007) also featured costs for Chile and Uruguay, two high-income countries. Only costs for Brazil, an upper-middle-income country, were included in the analysis

**Table 4:** Societal cost estimates by article (2018 US$). Mean and median estimates provided by different sets of costs within an article are reported as a range with the differences in estimates explained in the description

Source (author, year)	Mean (or median^a^) estimates of societal costs				Description of difference in estimates
	Direct medical	Direct non-medical	Indirect	Overall	
**Gastroenteritis**					
Indonesia, lower-middle-income country in Gavi accelerated transition					
Hospitalized					
Wilopo (2009) [22]	$42.16–$86.27	$5.56–$15.34	$6.88–$10.15	$54.61–$111.78	Primary vs secondary healthcare facilities; public vs private
Ambulatory					
Wilopo (2009) [22]	$5.90–$16.15	$0.31–$1.63	$1.22–$4.10	$6.42–$21.89	Primary vs secondary healthcare facilities
Kyrgyzstan, lower-middle-income country in Gavi preparatory transition					
Hospitalized					
Flem (2009) [21]	–	–	–	$78.12	
Vietnam, upper-middle-income country and not Gavi-eligible					
Hospitalized					
Fischer (2005) [36]	$35.79–$55.12	$8.89–$9.64	$8.81–$18.39	$63.07–$73.57	Urban vs rural settings
Ambulatory					
Fischer (2005) [36]	$5.86–$8.36	$0.81–$2.75	$2.26–$8.69	$11.60–$17.19	Secondary hospital vs primary healthcare clinic; public vs private
Not distinguished					
Riewpaiboon (2016) [70]	$34.89–$130.93	$13.14–$29.00	$43.31–$82.74	$91.90–$242.42	Rotavirus vs non-rotavirus; different age groups
**Pneumonia**					
Bangladesh, lower-middle-income country and in Gavi preparatory transition					
Hospitalized					
Ashraf (2010) [42]	$310.28	–	–	–	
Ambulatory					
Ashraf (2010) [42]	$198.72	–	–	–	
Colombia, upper-middle-income country and not Gavi-eligible					
Hospitalized					
Guzmán (2005) [48]	$709.76–$971.37	–	$116.65–$144.09	–	Viral vs bacterial illness
Fiji, upper-middle-income country and not Gavi-eligible					
Ambulatory					
Temple (2012) [27]	–	–	–	$15.21–$25.13	Tertiary hospital vs primary healthcare clinic
Gambia, low-income country and in Gavi initial self-financing					
Hospitalized					
Usuf (2016) [39]	–	–	–	$101.49	
Ambulatory					
Usuf (2016) [39]	–	–	–	$14.39	
Pakistan, lower-middle-income country in Gavi preparatory transition					
Hospitalized					
Hussain (2008) [35]	–	–	–	$108.95–$249.19	Severe vs very severe illness
Ambulatory					
Hussain (2008) [35]	–	–	–	$39.45	
Vietnam, upper-middle-income country and not Gavi-eligible					
Not distinguished					
Le (2014) [37]	–	–	–	$365.83	
**Meningitis**					
Gambia, low-income country and in Gavi initial self-financing					
Not distinguished					
Usuf (2016) [39]	–	–	–	$159.16	
Russian Federation, upper-middle-income country and not Gavi-eligible					
Not distinguished					
Platonov (2006) [31]	$3062	–	$172.41	$3405.04	
Vietnam, upper-middle-income country and not Gavi-eligible					
Not distinguished					
Le (2014) [37]	–	–	–	$834.35	

There were no costs from the societal perspective for influenza, Japanese encephalitis, hepatitis B, measles, yellow fever, or rubella. Country income statuses defined by the World Bank lending groups in 2020 [3]. Gavi status defined by the 2018 Gavi Annual Progress Report [13]

^a^Article reported median costs instead of mean costs

In addition to differences by the type of care provided, government costs per hospitalized episode increased with higher country income status (Table 3). Governments spent $46.76–$84.95, $130.86–$442.54, and $205.07–$6623.99 per hospitalized episode across all diseases in low-, lower-middle-, and upper-middle-income countries, respectively. In most articles including both the household and the government perspectives, governments faced greater costs per episode of illness than households. In Le et al., the government spent more than households on medical care for an episode of pneumonia and meningitis in Vietnam; however, when including non-medical costs, the household direct costs exceeded those of the government [37]. There were strong differences in societal costs across diseases and types of care (Table 4).

We examined the share of the costs from the household perspective and focused on 27 sets of costs (from nine articles) that looked at households using public healthcare facilities, who reported direct medical, direct non-medical, and indirect costs (Appendix 2 in ESM). Across diseases and settings, the proportion of non-medical and indirect costs outweighs the medical costs. Non-medical costs dominate (54%) other costs over outpatient care, while indirect costs take the highest share (43%) of the total cost for inpatient care. Medical costs made 21% of the cost of hospitalized pneumonia and between 1% and 14% for outpatient pneumonia, while non-medical and indirect costs estimated at 62–86% and 10–15%, respectively. For GE, medical costs made 12–44% of the cost of a hospitalized case with non-medical (14–16%) and indirect costs (41–72%). One set reported that households had no out-of-pocket expenses for outpatient GE, facing only indirect costs.

Three sets of costs included all three types of costs for households using private healthcare facilities, representing inpatient care only. The average proportion of direct medical costs (69%) is higher than that of direct non-medical (16%) and indirect costs (15%) (Appendix 2 in ESM).

### Economic Burden on Households

In addition to the breakdown of the burden of costs borne by the households, we examined whether authors took the additional step to interpret COI estimates in relation to income or total expenditure of the household or government, which we defined as economic burden measures.

In this review, 18 articles (60%) described the economic burden of illness with 45 economic burden measures. These composite measures contain a variety of numerators and denominators as demonstrated in Table 5.

**Table 5:** Types of numerators and denominators for economic burden measures

Article (first author, year)	Numerator as % of denominator	Numerator (primary data from articles)		Denominator		
		Costs included	Summary statistic	Type of cost	Summary statistic	Source of data
Jacob (2016) [16]	11%	Inpatient cost	Median	Household income	Median annual reported income	Primary
	1%	Outpatient cost	Median	Household income	Median annual reported income	Primary
Usuf (2016) [39]	Description: “one to ten times [the denominator]”	Inpatient cost	Mean	Household expenditure	Mean daily household expenditure	Secondary
Ngabo (2016) [17]	Average 21–110%	All costs	Total	Household income	Total monthly reported income	Primary
Loganathan (2015) [32]	0.39–23.20%	Out-of-pocket expenditure	Mean	Household income	Average monthly reported income	Primary
	5–20%	Direct and indirect cost	Mean	Household income	Mean monthly reported income	Primary
Soltani (2015) [67]	75%	Inpatient cost	Mean	Household income	Mean monthly reported income	Primary
Alkoshi (2015) [38]	7%	Indirect cost	Mean	Gov expenditure on health	Mean monthly	Secondary
Burke (2014) [18]	Average 1.40–2.20%	Direct and indirect cost	Total	Household income	Total annual reported income	Primary
Le (2014) [37]	43%	Out-of-pocket expenditure	Mean	Household expenditure	Total monthly reported expenditure	Both
	83%	Out-of-pocket expenditure	Mean	Household expenditure	Total monthly expenditure	N/S
Zhou (2013) [68]	41%	Direct medical cost	Mean	Household income	Mean annual GDP per capita	Secondary
	5.20%	Inpatient cost	Mean	Household income	Mean annual GDP per capita	Secondary
Burke (2013) [19]	Average 1–38.17%	Direct and indirect cost	Total	Household income	Total annual reported income	Primary
Sinha (2012) [26]	Up to 8%	Out-of-pocket expenditure	Mean	Household income	Median individual monthly income	Secondary
Temple (2012) [27]	Description: comparison with the basic needs poverty line and % of population under the poverty line	Outpatient cost	Mean	Household estimated income (basic need of adult under poverty line)	Mean weekly income	Secondary
MacIntyre (2010) [24]	Description: “[numerator] could represents > 10% of the [denominator]”	Out-of-pocket expenditure	Mean	Household income	Mean income of the community	Secondary
Chai (2009) [30]	20–26%	Out-of-pocket expenditure	Mean	Household income	Mean monthly reported income	Primary
Tate (2009) [20]	30%	Direct medical cost	Mean	Household income	Mean reported income	Secondary
	30%	Out-of-pocket expenditure	Mean	Household income	Mean reported income	Secondary
Chola (2009) [23]	Description: “[numerator] is higher than [denominator]”	Inpatient cost	Mean	Gov expenditure on health	Mean annual expenditure per capita	Secondary
Griffiths (2013) [15]	460–1000%	Out-of-pocket expenditure	Median	Household income	Median monthly reported income	Primary
Hussain (2006) [29]	82%	Outpatient cost	Mean	Gov health expenditure	Mean annual expenditure per capita	N/S

*GDP* gross domestic product, *Gov* government, *N/S* not specified

^a^If it is a percentage, the economic burden indicator is the numerator being X % of the denominator. If a description, then the indicator is a qualitative assessment

The result shows that the percentage of COI as a percentage of household expenditure ranges from 43% to 83%. Cost of illness as a percentage of household income ranges from 0.39% to 1000%, depending on the illness and the choice of numerator and denominator. Cost of illness as a percentage of government per capita expenditure falls between 3.5 and 82%.

### Funding

The main source of funding for the studies identified in this review came from multi-lateral agencies (WHO, UNICEF), non-government organizations, and philanthropies (22 articles, 59%), followed by governments and public organizations (16 articles, 43%) and the private sector (eight articles, 22%). Four articles did not disclose any source of funding [27, 28, 34, 35].

## Discussion

From 2000 to 2016, only 37 articles were found to produce COI estimates based on primary data collection for potentially pediatric VPD. Clabaugh and Ward [4] found 52 articles focusing on COI in the USA published between 2000 and 2004 collecting data from insurance claims and facility administration databases, including charges applied to patients and caregivers. Akobundu et al. [5] found 365 articles presenting COI estimates generated from primary data collection or by modeling existing cost estimates from the literature between 1996 and 2005. Their review included 20 articles focusing on LMIC, albeit none of which fulfilled the selection criteria for our review: different diseases of interest (epilepsy, malaria, human immunodeficiency virus [HIV]) and/or using modeled data. The scarcity of electronic and standardized patients’ records in LMIC implies a heavy reliance on a prospective approach to costing, implying that the researchers must conduct their own data collection—understandingly, an expensive endeavor.

The cost estimates for different illnesses were challenging to compare owing to the wide range of healthcare costs included, diverse disease definitions, and unclear perspectives. Building upon the work of Rice [41] and Hodgson and Meiners [7], Clabaugh and Ward made several recommendations to improve the reliability of COI studies [4]. In our selection of COI studies, most of these recommendations were met and we review here what is still missing.

*Study perspective* in their review, Clabaugh and Ward [4] first recommended to disclose the economic perspective considered and ensure its coherence with the costs included in the COI estimate. There was one article where the costs reported do not correspond with the study perspective [42]. The societal perspective should combine both the provider and the caregiver’s perspectives, and include indirect costs related to productivity loss.

*Definition and comprehensiveness of cost components* in parallel, researchers must identify and include all the components of care relevant to the treatment of the illness [4, 41]. All studies integrated the most tangible costs related to care such as medications, diagnostic tests, and non-medical costs. Two studies distinguished themselves by including capital and overhead costs [18, 29]. In Burke et al. [18], the researchers suggested that the real COI falls between the costs in the public sector and the costs from the private clinics, as the cost estimates for the former did not include administration costs, operation costs, and the depreciation of the infrastructure. While capital and overhead costs are only a small share of the COI estimate, they are not negligible.

Additionally, national guidelines provide strict rules for the care of these diseases, but local practices may diverge because of a lack of resources or oversight [43]. In most of the selected articles, item costs and utilization were drawn from medical health records and interviews, providing an accurate perspective on the ‘real-world’ COI. However, none of the articles explicitly discussed whether the care provided corresponded to the guidelines. Mathew et al. [44] mentioned that an increase in COI is linked to increased quality of care, yet no article explored whether lower COI is related to worse health outcomes. To understand how healthcare is provisioned and organized, researchers should conduct a brief qualitative study or consultations that complement quantitative data. While most recognized missing or unforeseen costs as a limitation, none of the selected articles reported conducting such a study proactively.

*Disease definition* Clabaugh and Ward [4] expressed the need to standardize the use of the “second” diagnosis received by a patient to confirm the case and its inclusion in the COI study. As opposed to the “first” diagnosis performed through the initial clinical assessment, the second diagnosis usually follows additional laboratory investigations and is, therefore, more reliable. Laboratory testing allows for more stringent eligibility criteria to be applied, where only cases with a specific disease etiology are included. Such a narrow disease definition may allow researchers to associate the economic data with specific interventions like vaccines. Over half of the articles used laboratory tests and radiology to ascertain the etiology of the disease.

The presence of comorbidities, particularly HIV/AIDS or malaria, is scarcely discussed in the selected articles. The articles were not clear on whether researchers included cases of children who had comorbidities or were immunocompromised. Only two articles [26, 45] focused on HIV and examined the differences in costs between immunocompromised and immunocompetent patients for acute lower respiratory tract infections. Note, however, that our review excluded articles that focused on HIV solely or on HIV and other infectious diseases than the nine diseases of interest.

*Time horizon* once the disease case is identified, the scope of the costs and the reliability of their reporting depends on the chosen time horizon [8]. All the selected articles opted for an incidence-based approach, associating the COI estimate to an episode of the illness rather than to a yearly average. The lack of studies taking a prevalence-based approach is not surprising as we focused on infectious illnesses characterized by the presence of an acute phase requiring treatment. It is possible to capture entire episodes of the illness even with such short periods for data collection. Furthermore, eight articles followed the patient beyond the acute phase of the illness. Doing so allowed the researchers to review the COI estimates reported during the initial interview and consolidate them with costs that occurred thereafter. Follow-ups can reduce recall bias and help obtain a more comprehensive set of the costs borne by the household. This said, only two articles estimated the cost of disabilities due to illness beyond the facility visit [31, 39].

*Source of data* As a good practice, authors should disclose the source of the data and how they combined data from different sources to generate cost estimates [4], particularly when complementing their data with estimates from secondary sources like WHO-CHOICE, as seen in Sinha et al. [26]. Furthermore, when adopting a retrospective approach, researchers should use as much as possible publicly available datasets to allow other researchers to replicate the research process [4]. Framed as part of a workshop or produced through a co-creative process, accessible inputs would find use in local policymaking and budget planning [41].

### Applications for Cost Data: Economic Burden of Illness

Estimating the economic burden of illness on households through catastrophic health expenditures is an official sustainable development goal indicator for monitoring the progress of financial protection provided by the universal health coverage [46]. Given the importance of economic burden measures for the global health agenda, we examined the studies selected for this systematic review to determine whether they took the additional step to measure the economic burden of illness on bearers of the costs. While 18 studies (60%) contain some measures of the economic burden of illness, 15 studies reported household income or expenditure as the denominator. Only eight of 15 studies provided estimates from primary data collection.

Beyond simply comparing the numerator against the denominator, several studies contain in-depth analyses of the difference in out-of-pocket household expenditure across income quintiles. Ngabo et al. [17] demonstrated the disproportionate impact of the pediatric inpatient admission for GE on the lowest quintile (110%) compared to the highest quintile (21%) in Rwanda [17]. Similarly, Loganathan et al. [32] found that out-of-pocket expenditure of the lowest quintile as a proportion of monthly household income (23.2%) was higher than that of the highest income quintile in Malaysia (5.7%). They also demonstrated that out-of-pocket costs were concentrated among the wealthy [32] based on poverty headcounts.

Furthermore, Burke et al. [18] developed a logistic regression model and identified potential predictors of catastrophic costs such as outpatient status, care seeking at a private hospital, whether treatment was previously sought for the GE episode, and the number of days of experiencing GE before the current visit.

These findings show that additional data on the distribution of income will provide further insights into the economic burden of illness across quintiles and the implications for increasing equity across population subgroups. Future COI studies will benefit from incorporating economic burden-related variables into primary data collection, which will help improve the application of COI estimates to health sector priority setting and budget planning as well as to the advancement of universal health coverage.

### Limitations of this Systematic Review

Considering their value for health technology assessment and program evaluation, the economic burden of illness is likely more frequently assessed than the literature suggests and with varying rigor, as our review suggests. It is not surprising to find COI studies integrated into randomized controlled trials and cost-effectiveness analyses. With our choice of keywords, we chose to capture a significant amount of studies, most of them irrelevant to COI, for the chance to find those hiding any assessments of the COI in their analysis. Notably, we cannot exclude human error from such a long screening process, and we may have missed some articles. Existing systematic reviews helped in limiting this risk [47]. It is possible that language played a role in limiting the search results, especially for articles published in Russian, Japanese, and Chinese: in our selection, one study was published in a regional journal and was not translated to English [48]. This said, with the exclusion of the gray literature and with the coverage of English, French, Spanish, and Portuguese, we believed that we were able to effectively limit this risk.

Another limitation may come from bringing the reported costs to a comparable currency (2018 US$). The inflation correction and the currency conversion may be distorting the COI estimates, particularly those from older articles. Open access to the dataset with raw and corrected data can allow researchers to change the corrections or use the original estimates [9].

Finally, we conducted a brief scoping review of the literature from January 2017 to May 2020 and found several articles that may have been eligible for inclusion post-2016. Particularly, we found two studies assessing the cost of measles in the Federated States of Micronesia [49] and the cost of measles and rubella in Romania [50] — a first for measles and rubella in LMIC. Other studies examined further the cost of GE [51–59], pneumonia [51, 52, 59–63], and influenza [64, 65]. Some of them generated COI estimates from a programmatic approach, evaluating the cost of treating diarrhea and pneumonia through an integrated community case management [51, 52, 59]. Such an approach generates questions on the representativeness of the COI estimates, particularly for regions where integrated community case management is not implemented, where alternative care processes are in place, or where it faces logistical shortcomings, such as medication stock-outs, that affect the COI and how people decide to procure healthcare [66].

### Implications for Policy and Research Priority Setting

Our review demonstrates that VPDs represent a significant economic burden both to households and the healthcare system. As COI data become more standardized and are more readily available for different settings, governments or stakeholder organizations will be able to directly compare the economic and financial burden of illnesses and develop policy targets and priorities accordingly. Within the health sector, decision makers can also apply COI data to better understand the financial realities of service utilization for different illnesses and better target interventions focused on improving the equity of healthcare access and utilization. Outside of the health sector, COI data can be used to understand how targeted investments in health may improve household economic well-being or generate spillover gains to the broader economy by freeing up disposable income for savings and non-health consumption, reducing catastrophic health expenditures, and improving labor force participation and productivity.

Using COI to generate transparent monetary assessments can also assist in the reframing of public health spending as investments that reduce incurred costs, rather than as pure costs. Such a reframing can also help to create parity in both languages and in impacts measured for investments in health vs in other sectors that seek to target directly financial and economic growth. The result may be a more direct discussion of health investments as a component of national strategic planning for economic growth and development more broadly.

## Conclusions

Data were extracted for 37 COI studies conducted on childhood VPD in LMIC, generating a total of 267 different sets of costs. The methodological heterogeneity across studies limited our ability to aggregate and compare costs. The lack of COI studies in LMIC with primary data collection for measles, hepatitis B, rubella, and YF was not surprising, considering the long-standing existence of vaccines that target these diseases. However, the resurgence of these diseases and the global interest towards eradication should motivate the development of COI estimates to assess the impact of such scenarios on healthcare budgets and households.

## Electronic supplementary material

Below is the link to the electronic supplementary material.

Supplementary file 1 (PDF 110 kb)

Supplementary file 2 (PDF 133 kb)

Supplementary file 3 (DOCX 49 kb)

Supplementary file 4 (PDF 104 kb)

## Data Availability

This study can be replicated with the dataset available in open access on DataVerse with the identifiers 10.7910/dvn/cb6x8k and at: https://dataverse.harvard.edu/dataverse/dove-coi-sr/.

## References

[1] Byford S, Torgerson DJ, Raftery J (2000). Economic note: cost of illness studies. BMJ.

[2] Jo C (2014). Cost-of-illness studies: concepts, scopes, and methods. Clin Mol Hepatol..

[3] World Bank. World Bank country and lending groups. Country classification. 2020. https://datahelpdesk.worldbank.org/knowledgebase/articles/906519-world-bank-country-and-lending-groups. Accessed 13 May 2020.

[4] Clabaugh G, Ward MM (2008). Cost-of-illness studies in the United States: a systematic review of methodologies used for direct cost. Value Health..

[5] Akobundu E, Ju J, Blatt L, Mullins CD (2006). Cost-of-illness Studies. Pharmacoeconomics..

[6] Moher D, Liberati A, Tetzlaff J, Altman DG, PRISMA Group (2009). Preferred Reporting Items for Systematic Reviews and Meta-Analyses: the PRISMA statement. PLoS Med..

[7] Hodgson TA, Meiners MR (1982). Cost-of-illness methodology: a guide to current practices and procedures. Milbank Mem Fund Q Health Soc..

[8] Vassall A, Sweeney S, Kahn J, Gomez GB, Bollinger L, Marseille, et al. Reference case for estimating the costs of global health services and interventions. Project Report. Global Health Cost Consortium; 2017. https://researchonline.lshtm.ac.uk/id/eprint/4653001 Accessed 17 July 2020.

[9] de Broucker G, Sim SY, Brenzel L, Gross M, Patenaude B, Constenla D. Replication data for: the cost of nine pediatric infectious illnesses in low- and middle-income countries: a systematic review of cost of illness studies. V2 ed. Baltimore: Harvard Dataverse; 2020. 10.1007/s40273-020-00940-4 PMC7578143 32748334

[10] World Bank. Official exchange rate (LCU per US$, period average). International Monetary Fund, International Financial Statistics. 2019. https://data.worldbank.org/indicator/PA.NUS.FCRF. Accessed 23 Sept 2019.

[11] World Bank. Consumer price index. International Monetary Fund, International Financial Statistics. 2019. https://data.worldbank.org/indicator/FP.CPI.TOTL. Accessed 23 Sept 2019.

[12] Viera AJ, Garrett JM (2005). Understanding interobserver agreement: the kappa statistic. Fam Med.

[13] Gavi (2018). Annual progress report.

[14] Bar-Zeev N, Tate JE, Pecenka C, Chikafa J, Mvula H, Wachepa R (2016). Cost-effectiveness of monovalent rotavirus vaccination of infants in Malawi: a postintroduction analysis using individual patient-level costing data. Clin Infect Dis.

[15] Griffiths MJ, Lemon JV, Rayamajhi A, Poudel P, Shrestha P, Srivastav V (2013). The functional, social and economic impact of acute encephalitis syndrome in Nepal: a longitudinal follow-up study. PLoS Neglect Trop Dis..

[16] Jacob J, Joseph TK, Srinivasan R, Kompithra RZ, Simon A, Kang G (2016). Direct and indirect costs of pediatric gastroenteritis in Vellore. India. Indian Pediatr..

[17] Ngabo F, Mvundura M, Gazley L, Gatera M, Rugambwa C, Kayonga E (2016). The economic burden attributable to a child’s inpatient admission for diarrheal disease in Rwanda. PLoS One.

[18] Burke RM, Smith ER, Dahl RM, Rebolledo PA, Calderón MEC, Cañipa (2014). The economic burden of pediatric gastroenteritis to Bolivian families: a cross-sectional study of correlates of catastrophic cost and overall cost burden. BMC Public Health..

[19] Burke RM, Rebolledo PA, Embrey SR, Wagner LD, Cowden CL, Kelly FM (2013). The burden of pediatric diarrhea: a cross-sectional study of incurred costs and perceptions of cost among Bolivian families. BMC Public Health..

[20] Tate JE, Chitambar S, Esposito DH, Sarkar R, Gladstone B, Ramani S (2009). Disease and economic burden of rotavirus diarrhoea in India. Vaccine..

[21] Flem ET, Latipov R, Nurmatov ZS, Xue Y, Kasymbekova KT, Rheingans RD (2009). Costs of diarrheal disease and the cost-effectiveness of a rotavirus vaccination program in Kyrgyzstan. J Infect Dis.

[22] Wilopo SA, Kilgore P, Kosen S, Soenarto Y, Aminah S, Cahyono (2009). Economic evaluation of a routine rotavirus vaccination programme in Indonesia. Vaccine..

[23] Chola L, Robberstad B (2009). Estimating average inpatient and outpatient costs and childhood pneumonia and diarrhoea treatment costs in an urban health centre in Zambia. Cost Eff Resour Alloc..

[24] MacIntyre UE, De Villiers FPR (2010). The economic burden of diarrheal disease in a tertiary level hospital, Gauteng. South Africa. Journal of Infectious Diseases..

[25] Anh DD, Riewpaiboon A, Tho H, Kim SA, Nyambat B, Kilgore P (2010). Treatment costs of pneumonia, meningitis, sepsis, and other diseases among hospitalized children in Viet Nam. J Health Popul Nutr..

[26] Sinha A, Kim S, Ginsberg G, Franklin H, Kohberger R, Strutton D (2012). Economic burden of acute lower respiratory tract infection in South African children. Paediatr Int Child Health..

[27] Temple B, Griffiths UK, Mulholland EK, Ratu FT, Tikoduadua L, Russell FM (2012). The cost of outpatient pneumonia in children < 5 years of age in Fiji. Trop Med Int Health..

[28] Lee WS, Poo MI, Nagaraj S (2007). Estimates of economic burden of providing inpatient care in childhood rotavirus gastroenteritis from Malaysia. J Paediatr Child Health.

[29] Hussain H, Waters H, Omer SB, Khan A, Baig IY, Mistry R (2006). The cost of treatment for child pneumonias and meningitis in the Northern Areas of Pakistan. Int J Health Plann Manage..

[30] Chai PF, Lee WS (2009). Out-of-pocket costs associated with rotavirus gastroenteritis requiring hospitalization in Malaysia. Vaccine..

[31] Platonov AE, Griffiths UK, Voeykova MV, Platonova OV, Shakhanina IL, Chistyakova GG (2006). Economic evaluation of Haemophilus influenzae type b vaccination in Moscow. Russian Federation. Vaccine..

[32] Loganathan T, Lee WS, Lee KF, Jit M, Ng CW (2015). Household catastrophic healthcare expenditure and impoverishment due to rotavirus gastroenteritis requiring hospitalization in Malaysia. PLoS One.

[33] Tate JE, Rheingans RD, Oreilly CE, Obonyo B, Burton DC, Tornheim JA (2009). Rotavirus disease burden and impact and cost- effectiveness of a rotavirus vaccination program in Kenya. J Infect Dis.

[34] Madsen HO, Hanehøj M, Das AR, Moses PD, Rose W, Puliyel M (2009). Costing of severe pneumonia in hospitalized infants and children aged 2-36 months, at a secondary and tertiary level hospital of a not-for-profit organization. Trop Med Int Health..

[35] Hussain H, Waters H, Khan AJ, Omer SB, Halsey NA (2008). Economic analysis of childhood pneumonia in Northern Pakistan. Health Policy Plan..

[36] Fischer TK, Anh DD, Antil L, Cat NDL, Kilgore PE, Thiem VD (2005). Health care costs of diarrheal disease and estimates of the cost-effectiveness of rotavirus vaccination in Vietnam. J Infect Dis.

[37] Le P, Griffiths UK, Anh DD, Franzini L, Chan W, Pham H (2014). The economic burden of pneumonia and meningitis among children less than five years old in Hanoi. Vietnam. Trop Med Int Health..

[38] Alkoshi S, Leshem E, Parashar UD, Dahlui M (2015). Anticipating rotavirus vaccines: a pre-vaccine assessment of incidence and economic burden of rotavirus hospitalizations among children < 5 year of age in Libya, 2012-13. BMC Public Health..

[39] Usuf E, Mackenzie G, Sambou S, Atherly D, Suraratdecha C (2016). The economic burden of childhood pneumococcal diseases in The Gambia. Cost Eff Resour Alloc..

[40] Patel AB, Bang A, Singh M, Dhande L, Chelliah LR, Malik A (2015). A randomized controlled trial of hospital versus home based therapy with oral amoxicillin for severe pneumonia in children aged 3–59 months: the IndiaCLEN Severe Pneumonia Oral Therapy (ISPOT) Study. BMC Pediatr..

[41] Rice DP (1967). Estimating the cost of illness. Am J Public Health Nat Health.

[42] Ashraf H, Mahmud R, Alam NH, Jahan SA, Kamal SM, Haque F (2010). Randomized controlled trial of day care versus hospital care of severe pneumonia in Bangladesh. Pediatrics.

[43] Ayieko P, Irimu G, Ogero M, Mwaniki P, Malla L, Julius T (2019). Effect of enhancing audit and feedback on uptake of childhood pneumonia treatment policy in hospitals that are part of a clinical network: a cluster randomized trial. Implement Sci..

[44] Mathew A, Srinivasan R, Venugopal S, Kang G (2016). Direct medical costs in children with rotavirus and non-rotavirus diarrhea admitted to a pediatric intensive care unit and high dependency unit in Delhi. Indian Pediatr.

[45] Kitchin OP, Wessels F, Masekela R, Becker P, Green RJ (2011). Costs of admission for paediatric pneumonia in a setting of human immunodeficiency virus infection. Int J tuberculosis Lung Dis..

[46] World Health Organization, International Bank for Reconstruction and Development/The World Bank (2017). Tracking universal health coverage: 2017 global monitoring report.

[47] Zhang S, Sammon PM, King I, Andrade AL, Toscano CM, Araujo SN (2016). Cost of management of severe pneumonia in young children: systematic analysis. J Glob Health..

[48] Guzmán NA, De La Hoz Restrepo F, Higuera AB, Pastor D, Di Fabio JL (2005). The economic costs of pneumonia in children under 2 years of age in Colombia. Rev Panam Salud Publica.

[49] Pike J, Tippins A, Nyaku M, Eckert M, Helgenberger L, Underwood JM (2017). Cost of a measles outbreak in a remote island economy: 2014 Federated States of Micronesia measles outbreak. Vaccine..

[50] Njau J, Janta D, Stanescu A, Pallas SS, Pistol A, Khetsuriani N (2019). Assessment of economic burden of concurrent measles and rubella outbreaks, Romania, 2011-2012. Emerg Infect Dis.

[51] Daviaud E, Besada D, Leon N, Rohde S, Sanders D, Oliphant N (2017). Costs of implementing integrated community case management (iCCM) in six African countries: implications for sustainability. J Glob Health..

[52] Escribano Ferrer B, Hansen KS, Gyapong M, Bruce J, Narh Bana SA, Narh CT (2017). Cost-effectiveness analysis of the national implementation of integrated community case management and community-based health planning and services in Ghana for the treatment of malaria, diarrhoea and pneumonia. Malar J..

[53] Halder AK, Luby SP, Akhter S, Ghosh PK, Johnston RB, Unicomb L (2017). Incidences and costs of illness for diarrhea and acute respiratory infections for children < 5 years of age in rural Bangladesh. Am J Trop Med Hyg.

[54] Hendrix N, Bar-Zeev N, Atherly D, Chikafa J, Mvula H, Wachepa R (2017). The economic impact of childhood acute gastroenteritis on Malawian families and the healthcare system. BMJ Open.

[55] Koksal T, Akelma AZ, Koksal AO, Kutukoglu I, Ozdemir O, Yuksel CN (2017). Cost-effectiveness of rotavirus vaccination in Turkey. J Microbiol Immunol Infect.

[56] Kukla M, McKay N, Rheingans R, Harman J, Schumacher J, Kotloff KL (2017). The effect of costs on Kenyan households’ demand for medical care: why time and distance matter. Health Policy Plan..

[57] Nonvignon J, Atherly D, Pecenka C, Aikins M, Gazley L, Groman D (2018). Cost-effectiveness of rotavirus vaccination in Ghana: examining impacts from 2012 to 2031. Vaccine..

[58] Rochanathimoke O, Riewpaiboon A, Tharmaphornpilas P, Jiamsiri S, Thavorncharoensap M, Postma MJ (2019). Economic burden of rotavirus diarrhea in Thailand: rfrom a pilot study on rotavirus vaccination. Vaccine..

[59] Soremekun S, Kasteng F, Lingam R, Vassall A, Kertho E, Settumba S (2018). Variation in the quality and out-of-pocket cost of treatment for childhood malaria, diarrhoea, and pneumonia: community and facility based care in rural Uganda. PLoS One.

[60] Andrade AL, Afonso ET, Minamisava R, Bierrenbach AL, Cristo EB, Morais-Neto OL (2017). Direct and indirect impact of 10-valent pneumococcal conjugate vaccine introduction on pneumonia hospitalizations and economic burden in all age-groups in Brazil: a time-series analysis. PLoS One.

[61] Ezoji K, Yaghoubi M, Nojomi M, Mahmoody S, Zahraie SM, Moradi-Lakeh M (2019). Cost-effectiveness of introducing the pneumococcal conjugate vaccine for children under 5 years in the Islamic Republic of Iran. East Mediterr Health J..

[62] Kebede TT, Svensson M, Addissie A, Trollfors B, Andersson R (2019). Cost-effectiveness of childhood pneumococcal vaccination program in Ethiopia: results from a quasi-experimental evaluation. BMC Public Health..

[63] Shen K, Wasserman M, Liu D, Yang YH, Yang J, Guzauskas GF (2018). Estimating the cost-effectiveness of an infant 13-valent pneumococcal conjugate vaccine national immunization program in China. PLoS One.

[64] Orenstein EW, Orenstein LA, Diarra K, Djiteye M, Sidibe D, Haidara FC (2017). Cost-effectiveness of maternal influenza immunization in Bamako, Mali: a decision analysis. PLoS One.

[65] Kittikraisak W, Suntarattiwong P, Kanjanapattanakul W, Ditsungnoen D, Klungthong C, Lindblade KA (2018). Comparison of incidence and cost of influenza between healthy and high-risk children < 60 months old in Thailand, 2011-2015. PLoS One.

[66] Nanyonjo A, Counihan H, Siduda SG, Belay K, Sebikaari G, Tibenderana J (2019). Institutionalization of integrated community case management into national health systems in low- and middle-income countries: a scoping review of the literature. Glob Health Action..

[67] Soltani MS, Ben Salah A, Bouanene I, Trabelsi A, Sfar MT, Harbi A (2015). Epidemiology and medical cost of hospitalization due to rotavirus gastroenteritis among children under 5 years of age in the central-east of Tunisia. Eastern Med Health J..

[68] Zhou L, Situ S, Huang T, Hu S, Wang X, Zhu (2013). Direct medical cost of influenza-related hospitalizations among severe acute respiratory infections cases in three provinces in China. PLoS One..

[69] Constenla D (2007). Evaluating the costs of pneumococcal disease in selected Latin American countries. Rev Panam Salud Publica.

[70] Riewpaiboon A, Shin S, Le TP, Vu DT, Nguyen TH, Alexander N (2016). Cost of rotavirus diarrhea for programmatic evaluation of vaccination in Vietnam. BMC Public Health..

[71] Sadruddin S, Shehzad S, Bari A, Khan A, Ibad Ul H, Khan A (2012). Household costs for treatment of severe pneumonia in Pakistan. Am J Trop Med Hyg.

[72] Ayieko P, Akumu AO, Griffiths UK, English M (2009). The economic burden of inpatient paediatric care in Kenya: household and provider costs for treatment of pneumonia, malaria and meningitis. Cost Eff Resour Alloc..

