# Supplementary material for: Cost of Nine Pediatric Infectious Illnesses in Low- and Middle-Income Countries: A Systematic Review of Cost-of-Illness Studies
Source: Pharmacoeconomics. 2020 Aug 4;38(10):1071–94. doi: 10.1007/s40273-020-00940-4 (PMC7578143; doi:10.1007/s40273-020-00940-4)
Supplement: Supplementary file 1 — (PDF 110 kb) [file 40273_2020_940_MOESM1_ESM.pdf]

Appendix I

TABLE I.1: Criteria to differentiate the sets of costs.

Criteria	Main options	Description
**Country**	A country in a multi-country study	Each country has its settings, currency, and healthcare provision.
**Settings**	Urban and/or peri-urban<br>Rural<br>Mixed urban and rural	The settings may be a factor of influence on the availability of services and transportation. When the settings or healthcare facilities were not specified in the article, we assumed “mixed urban and rural”.
**Facility ownership**	Public<br>Private	Public and private facilities are financed and operated differently.
**Facility level**	Primary<br>Secondary<br>Tertiary	Primary, secondary, and tertiary facility levels offer different levels of services. However, most sets of costs combined several facility levels.
**Age group**	Reported age group	The age of the children treated may influence the type of healthcare services provided and whether the child is hospitalized or not for the same condition.
**Illness & severity**	A definition of a case/episode	COI is defined around an episode of the illness leading to seeking care.
**Type of care**	Inpatient care<br>Outpatient care<br>Undistinguished inpatient/outpatient	Patients that stayed for at least one night at the healthcare facility were counted as receiving inpatient care. Some sets included care received before hospitalization as outpatient care. If no distinction of the level of care for an episode of the illness was made (whether it was inpatient or outpatient care), then we labeled it as “undistinguished”.
**Economic perspective**	Household<br>Government<br>Private sector<br>Healthcare (mixed public/private)<br>Third-party payer<br>Societal	The choice of costs included in the COI estimates depended on the economic perspective taken. Government, private sector, and healthcare (mixed) were all considered as the “healthcare system perspective”. The third-party payer perspective mainly considered the health insurance’s point of view. Societal was usually an aggregation of the household’s and healthcare system’s perspectives.
**Other parameters**	Customized by reviewer	Researchers identified other parameters that influenced the COI estimates specifically for the settings where the investigation took place. These parameters are reported in the database.
