# Supplementary material for: Cost of Nine Pediatric Infectious Illnesses in Low- and Middle-Income Countries: A Systematic Review of Cost-of-Illness Studies
Source: Pharmacoeconomics. 2020 Aug 4;38(10):1071–94. doi: 10.1007/s40273-020-00940-4 (PMC7578143; doi:10.1007/s40273-020-00940-4)
Supplement: Supplementary file 2 — (PDF 133 kb) [file 40273_2020_940_MOESM2_ESM.pdf]

Appendix II

TABLE II.1: Average share of costs for households using public healthcare facilities and that reported all 3 types of costs by type of costs (unweighted).

	Average proportion of costs			Number of sets of costs
	Direct medical costs	Direct non-medical costs	Indirect costs	
**PNEUMONIA**	**22%**	**64%**	**14%**	**7**
**Urban**	**24%**	**62%**	**15%**	**6**
Inpatient care	21%	66%	13%	4
Outpatient care	1%	86%	13%	1
Not distinguished (inpatient/outpatient)	58%	19%	22%	1
**Mixed urban/rural**	**14%**	**76%**	**10%**	**1**
Outpatient care	14%	76%	10%	1
**GASTROENTERITIS**	**31%**	**16%**	**53%**	**19**
**Rural**	**12%**	**15%**	**72%**	**4**
Inpatient care	12%	15%	72%	4
**Urban**	**40%**	**17%**	**43%**	**12**
Inpatient care	44%	14%	41%	9
Not distinguished (inpatient/outpatient)	28%	25%	47%	3
**Mixed urban/rural**	**21%**	**11%**	**68%**	**3**
Inpatient care	32%	16%	52%	2
Outpatient care	0%	0%	100%	1
**MENINGITIS**	**36%**	**34%**	**30%**	**1**
**Urban**	**36%**	**34%**	**30%**	**1**
Not distinguished (inpatient/outpatient)	36%	34%	30%	1
**Overall**	**29%**	**29%**	**42%**	**27**
Inpatient care	31%	26%	43%	19
Outpatient care	5%	54%	41%	3
Not distinguished (inpatient/outpatient)	36%	26%	39%	5

TABLE II.2: Average share of costs for households using private healthcare facilities and that reported all 3 types of costs by type of costs (unweighted).

	Average proportion of costs			Number of sets
	Direct medical costs	Direct non-medical costs	Indirect costs	
**PNEUMONIA**	**74%**	**18%**	**8%**	**2**
**Urban**	74%	18%	8%	2
Inpatient care	74%	18%	8%	2
**GASTROENTERITIS**	**61%**	**11%**	**29%**	**1**
**Urban**	**61%**	**11%**	**29%**	**1**
Not distinguished (inpatient/outpatient)	61%	11%	29%	1
**Overall**	**69%**	**16%**	**15%**	**3**
Inpatient care	74%	18%	8%	2
Not distinguished (inpatient/outpatient)	61%	11%	29%	1
