# Supplementary material for: Cost of Nine Pediatric Infectious Illnesses in Low- and Middle-Income Countries: A Systematic Review of Cost-of-Illness Studies
Source: Pharmacoeconomics. 2020 Aug 4;38(10):1071–94. doi: 10.1007/s40273-020-00940-4 (PMC7578143; doi:10.1007/s40273-020-00940-4)
Supplement: Supplementary file 3 — (DOCX 49 kb) [file 40273_2020_940_MOESM3_ESM.docx]

# Appendix III

**Search strategy and keywords used:**

(Concept 1: Disease) AND (Concept 2: Children) AND (Concept 3: Cost) AND (Concept 4: LMIC) NOT (Concept 5: Animals)

Limits: 2000-2017

**PubMed**

**Concept 1: Disease**

"Buruli Ulcer"[Mesh] OR "Mycobacterium Infections"[Mesh] OR "Chagas Disease"[Mesh] OR "Cholera"[Mesh] OR "Chikungunya Fever"[Mesh] OR "Chikungunya virus"[Mesh] OR "Hemorrhagic Fever, Crimean"[Mesh] OR "Dengue"[Mesh] OR "Severe Dengue"[Mesh] OR "diarrhea"[MeSH] OR "dysentery"[MeSH] OR "gastrointestinal diseases"[MeSH] OR "Diphtheria"[Mesh] OR "Hemorrhagic Fever, Ebola"[Mesh] OR "Echinococcosis"[Mesh] OR "Ebolavirus"[Mesh] OR "Gastroenteritis"[Mesh] OR "Hepatitis A"[Mesh] OR "Hepatitis B"[Mesh] OR "Hepatitis B, Chronic"[Mesh] OR "Hepatitis C, Chronic"[Mesh] OR "Hepatitis C"[Mesh] OR "Hepatitis E"[Mesh] OR "Echinococcosis, Hepatic"[Mesh] OR "Echinococcosis, Pulmonary"[Mesh] OR "HIV"[Mesh] OR "HIV Infections"[MeSH] OR "Papillomaviridae"[Mesh] OR "Influenza, Human"[Mesh] OR "Encephalitis, Japanese"[Mesh] OR "Lassa Fever"[Mesh] OR "Leishmaniasis"[Mesh] OR "Elephantiasis, Filarial"[Mesh] OR "Malaria"[Mesh] OR "Marburg Virus Disease"[Mesh] OR "Neisseria meningitidis"[Mesh] OR "Meningococcal Infections"[Mesh] OR "Meningitis, Meningococcal"[Mesh] OR "Measles"[Mesh] OR "Rubella"[Mesh] OR "Monkeypox"[Mesh] OR "Mumps"[Mesh] OR "Onchocerciasis"[Mesh] OR "Onchocerciasis"[Mesh] OR "Whooping Cough"[Mesh] OR "Plague"[Mesh] OR "Pneumococcal Infections"[Mesh] OR "Meningitis, Pneumococcal"[Mesh] OR "Pneumonia, Pneumococcal"[Mesh] OR "Poliomyelitis"[Mesh] OR "Rabies"[Mesh] OR "Rabies virus"[Mesh] OR "Rift Valley Fever"[Mesh] OR "Measles"[Mesh] OR "Rubella"[Mesh] OR "Smallpox"[Mesh] OR "Nematode Infections"[Mesh] OR "Taeniasis"[Mesh] OR "Cysticercosis"[Mesh] OR "Tetanus"[Mesh] OR "Trachoma"[Mesh] OR "Trypanosomiasis"[Mesh] OR "Tuberculosis"[Mesh] OR "Helminthiasis"[Mesh] OR "Ascaris lumbricoides"[Mesh] OR "Trypanosomiasis, African"[Mesh] OR "Herpesvirus 3, Human"[Mesh] OR "Chickenpox"[Mesh] OR "Yaws"[Mesh] OR "Yellow Fever"[Mesh] OR "Yellow fever virus"[Mesh] OR "Zika Virus"[Mesh] OR "Zika Virus Infection"[Mesh] OR "Leprosy"[Mesh] OR "Buruli Ulcer"[tw] OR " Mycobacterium ulcerans Infection"[tw] OR "Mycobacterium Infections"[tw] OR "Mycobacterium Infection"[tw] OR "Chagas"[tw] OR "Trypanosomiasis"[tw] OR "Cholera"[tw] OR "Choleras"[tw] OR "Chikungunya"[tw] OR "Crimean Hemorrhagic Fever"[tw] OR "Congo Virus Infections"[tw] OR "Crimean Hemorrhagic Fevers"[tw] OR "Congo Virus Infection"[tw] OR "Dengue"[tw] OR "Breakbone Fever"[tw] OR "Break bone Fever"[tw] OR "diarrhea"[tw] OR "diarrhoea"[tw] OR "diarrheal"[tw] OR "diarrhoeal"[tw] OR "dysentery"[tw] OR "Gastrointestinal Disease"[tw] OR "gastrointestinal diseases"[tw] OR "Diphtheria"[tw] OR "Diphtherias"[tw] OR "Dracunculiases"[tw] OR "Guinea Worm Infection"[tw] OR "Guinea Worm Infections"[tw] OR "Guinea Worm Disease"[tw] OR "Guinea Worm Diseases"[tw] OR "Dracunculosis"[tw] OR "Dracunculoses"[tw] OR "ebola"[tw] OR "Echinococcosis"[tw] OR "Echinococcoses"[tw] OR "Hydatidosis"[tw] OR "Hydatidoses"[tw] OR "Hepatic Echinococcoses"[tw] OR "Hydatid Cyst"[tw] OR "Hydatid Cysts"[tw] OR "Foodborne trematodiases"[tw] OR "Gastroenteritis"[tw] OR Gastroenteritide[tw] OR "Hepatitis A"[tw] OR "hep A"[tw] OR "Hepatitis B"[tw] OR "Hepatitis E"[tw] OR "Hepatitides"[tw] OR "ET-NANBH"[tw] OR "Acquired Immunodeficiency Syndrome"[tw] OR "human immunodeficiency virus"[tw] OR "human immune deficiency virus"[tw] OR HIV[tw] OR AIDS[tw] OR "Papillomaviridae"[tw] OR "Human Papilloma Virus"[tw] OR "Human Papilloma Viruses"[tw] OR "Human Papillomavirus"[tw] OR ((human[tw] OR humans[tw]) AND (Influenzas[tw] OR influenza[tw] OR flu[tw])) OR "Japanese B Viral Encephalitis"[tw] OR "Japanese B Encephalitis"[tw] OR "Japanese Encephalitis"[tw] OR "Lassa Fever"[tw] OR "Lassa Fevers"[tw] OR "Leishmaniasis"[tw] OR "Leprosy"[tw] OR "Leprosies"[tw] OR "Hansen Disease"[tw] OR "Hansen's Disease"[tw] OR "Hansens Disease"[tw] OR "Lymphatic filariasis"[tw] OR "Filarial Elephantiases"[tw] OR "Lymphatic Filariasis"[tw] OR "Filarial Elephantiasis"[tw] OR "Lymphatic Filariases"[tw] OR "Bancroftian Elephantiasis"[tw] OR "Bancroftian Elephantiases"[tw] OR "Malaria"[tw] OR "Marburg Virus Disease"[tw] OR "Marburg Disease"[tw] OR "Marburg Hemorrhagic Fever"[tw] OR "Meningococcal"[tw] OR "meningococci"[tw] OR "Meningococcus"[tw] OR "Micrococcus intracellularis"[tw] OR "Neisseria weichselbaumi"[tw] OR "meningitides"[tw] OR "Meningitis"[tw] OR "Measles"[tw] OR Rubeola[tw] OR "morbilli"[tw] OR "Rubella"[tw] OR "Rubellas"[tw] OR "epidemic roseola"[tw] OR "Monkeypox"[tw] OR "Monkey pox"[tw] OR "Mumps"[tw] OR "Parotitis"[tw] OR "parodotis"[tw] OR Onchocerciases[tw] OR "Whooping Cough"[tw] OR "Pertussis"[tw] OR "Pertusses"[tw] OR "Plague"[tw] OR "parotiditis"[tw] OR OR "Pneumococcal infections"[tw] OR "Pneumococcal infection"[tw] OR "pneumoniae Infections"[tw] OR "pneumoniae Infection"[tw] OR "Streptococcus pneumonia"[tw] OR "S.pneumoniae"[tw] OR "Poliomyelitis"[tw] OR "polio"[tw] or "poliovirus"[tw] OR "polios"[tw] OR "Infantile Paralysis"[tw] OR "Hubert disease"[tw] OR "lyssavirus"[tw] OR "lytta"[tw] OR "Hydrophobia"[tw] OR "Lyssa"[tw] OR "Lyssas"[tw] OR "Rift Valley Fever"[tw] OR "Measles"[tw] OR "Rubeola"[tw] OR "morbilli"[tw] OR "Rubella"[tw] OR "Rubellas"[tw] OR "epidemic roseola"[tw] OR "german measles"[tw] OR "Smallpox"[tw] OR "Variola"[tw] OR "Variolas"[tw] OR "Alastrim"[tw] OR "Helminthiasis"[tw] OR "Helminthiases"[tw] OR "Nematomorpha Infection"[tw] OR "Nematode Infections"[tw] OR "Nematode Infection"[tw] OR "Nematomorpha Infections"[tw] OR "soil transmitted Helminth infection"[tw] OR "soil transmitted Helminth infections"[tw] OR "Larva migrans syndrome"[tw] OR (("roundworm"[tw] OR Secernentea[tw] OR "whipworm"[tw] OR "Ascaris lumbricoides"[tw] OR "Ascaris lumbricoide"[tw] OR "Trichuris trichiura"[tw] OR "hookworms"[tw] OR "hookworm"[tw] OR "Necator americanus"[tw] OR "Ancylostoma duodenale"[tw] OR "Enterobius vermicularis"[tw]) AND ("infections"[tw] OR "infection"[tw])) OR "Taeniasis"[tiab] OR "Taeniases"[tw] OR "Taenia Infections"[tw] OR "Taenia Infection"[tw] OR "Cysticercosis"[tw] OR Cysticercoses[tw] OR "Tetanus"[tiab] OR "Trachoma"[tw] OR Trachomas[tw] OR "Egyptian Ophthalmia"[tw] OR "Trypanosomiasis"[tw] OR "Trypanosomiases"[tw] OR "African Trypanosomiases"[tw] OR "African Trypanosomiasis"[tw] OR "African Sleeping Sickness"[tw] OR "African Sleeping Sicknesses"[tw] OR "Nagana"[tw] OR "Tuberculosis"[tw] OR "Tuberculoses"[tw] OR "Kochs Disease"[tw] OR "Koch’s Disease"[tw] OR "Koch Disease"[tw] OR "Human Herpesvirus 3"[tw] OR "Chickenpox"[tw] OR "Chicken pox"[tw] OR "Herpes zoster"[tw] OR "HHV-3"[tw] OR "Varicella-Zoster"[tw] OR "Herpesvirus 3"[tw] OR "VZ Virus"[tw] OR "VZ Viruses"[tw] OR "Herpesvirus Varicellae"[tw] OR "varicella"[tw] OR "Yaws"[tw] OR Frambesia[tw] OR Frambesias[tw] OR "Yellow Fever"[tw] OR "Yellow Fevers"[tw] OR "Zika Virus"[tw] OR "Bairnsdale ulcer"[tw] OR "Buruli disease"[tw] OR "mycobacteriosis"[tw] OR "myobacteriosis”[tw] OR "Trypanosoma cruzi"[tw] OR "chickungunya"[tw] OR "cholera"[tw] OR "Congo Hemorrhagic Fevers"[tw] OR "dandy fever"[tw] OR "red fever"[tw] OR "solar fever"[tw] OR diphtheria[tw] OR "gastro enteritis"[tw] OR gastroduodenitis[tw] OR "gastrointestinal acute infection"[tw] OR "type a hepatitis"[tw] OR "Hepatitis type A"[tw] OR "botkin disease"[tw] OR "Hepatovirus"[tw] OR "type b Hepatitis"[tw] OR "Hepatitis type B"[tw] OR "non a non b hepatitis"[tw] OR "Hepatitis type B"[tw] OR "hydatid lung cyst"[tw] OR "lung echinococcal cyst"[tw] OR "lung echinococcosis"[tw] OR "lung echinococcus"[tw] OR "lung hydatid disease"[tw] OR "lung hydatidosis"[tw] OR "pulmonary echinococcal cyst"[tw] OR "pulmonary echinococcosis"[tw] OR "pulmonary Echinococcus cyst"[tw] OR "pulmonary hydatid cyst"[tw] OR "pulmonary hydatidosis"[tw] OR "human immunodeficiency virus"[tw] OR "human immuno deficiency virus"[tw] OR "immuno deficiency associated virus"[tw] OR "immunodeficiency associated virus"[tw] OR "human immune deficiency virus"[tw] OR "black fever"[tw] OR "leishmaniosis"[tw] OR "kala azar"[tw] OR "lymphatic filariasis"[tw] OR "filarial lymphedema"[tw] OR "filarial lymphooedema"[tw] OR "paludism"[tw] OR "Marburg syndrome"[tw] OR "Marburg disease"[tw] OR "Marburg virus disease"[tw] OR "meningococcic"[tw] OR meningococceal[tw] OR meningococcaemia[tw] OR "parotid inflammation"[tw] OR partitis[tw] OR Onchocerciases[tw] OR "Onchocerca infection"[tw] OR "Onchocerca volvulus infection"[tw] OR "Onchocerca infections"[tw] OR "Onchocerca volvulus infections"[tw] OR "onchocercosis"[tw] OR "onchocerosis"[tw] OR "Yersinia pestis"[tw] OR "Pneumococcal infections"[tw] OR "Pneumococcal infection"[tw] OR "pneumococci infection"[tw] OR "pneumococci infections"[tw] OR "pneumococcal disease"[tw] OR pneumococcosis[tw] OR "Small pox"[tw] OR "Chlamydia conjunctivitis"[tw] OR "Chlamydia trachomatis conjunctivitis"[tw] OR "chlamydial conjunctivitis"[tw] OR "granular conjunctivitis"[tw] OR "Trypanosoma infection"[tw] OR "trypanosome infection"[tw] OR "Trypanosoma infections"[tw] OR "trypanosome infections"[tw] OR framboesia[tw] OR parangi[tw] OR pian[tw] OR "Treponema pertenue infection"[tw]

**Concept 2: Children**

"Infant"[mesh] OR "Infant, Newborn"[mesh] OR "child"[mesh] OR "Child, Preschool"[Mesh] OR "Minors"[Mesh] OR "Adolescent"[Mesh] OR "Young Adult"[Mesh] OR "infant"[tw] OR "infants"[tw] OR "neonate"[tw] OR "neonates"[tw] OR "neonatal"[tw] OR "newborn"[tw] OR "newborns"[tw] OR "new-born"[tw] OR "new-borns"[tw] OR "baby"[tw] OR "babies"[tw] OR "Premature"[tw] OR "preterm"[tw] OR "pre term"[tw] OR "child"[tw] OR "children"[tw] OR "youth"[tw] OR "youths"[tw] OR "young people"[tw] OR "childhood"[tw] OR "toddler"[tw] OR "toddlers"[tw] OR "kid"[tw] OR "kids"[tw] OR "young patient"[tw] OR "young patients"[tw] OR "boy"[tw] OR "boys"[tw] OR "girl"[tw] OR "girls"[tw] OR "young age"[tw] OR "pediatric"[tw] OR "pre-schooler"[tw] OR "preschooler"[tw] OR "under 5"[tw] OR "under five"[tw] OR "under fives"[tw] OR "less than five"[tw] OR "Adolescents"[tw] OR "Adolescence"[tw] OR "Teens"[tw] OR "Teen"[tw] OR "Teenagers"[tw] OR "Teenager"[tw] OR "Youth"[tw] OR "Youths"[tw] OR "young adult"[tw] OR "young adults"[tw] OR "Minor"[tw] OR "Minors"[tw]

**Concept 3: Cost**

"Economics"[Mesh] OR "costs and cost analysis"[mesh] OR "cost-benefit analysis"[mesh] OR "cost control"[mesh] OR "cost of illness"[mesh] OR "Value of Life"[Mesh] OR "health care costs"[mesh] OR "Economics"[tw] OR "Economic"[tw] OR "pricing"[tw] OR cost[tw] OR Costs[tw] OR "Burden of Illness"[tw] OR "Illness Burden"[tw] OR "Illness Burdens"[tw] OR "value of life"[tw] OR "financial"[tw] OR "finance"[tw] OR "financing"[tw] OR "price"[tw] OR "prices"[tw] OR "market"[tw] OR "spending"[tw] OR "expenditure"[tw]

**Concept 4: LMIC**

("emerging country"[tiab] OR "emerging countries"[tiab] OR "emerging nation"[tiab] OR "emerging nations"[tiab] OR "emerging population"[tiab] OR "emerging populations"[tiab] OR "developing country"[tiab] OR "developing countries"[tiab] OR "developing nation"[tiab] OR "developing nations"[tiab] OR "developing population"[tiab] OR "developing populations"[tiab] OR "developing world"[tiab] OR "less developed country"[tiab] OR "less developed countries"[tiab] OR "less developed nation"[tiab] OR "less developed nations"[tiab] OR "less developed population"[tiab] OR "less developed populations"[tiab] OR "less developed world"[tiab] OR "lesser developed country"[tiab] OR "lesser developed countries"[tiab] OR "lesser developed nation"[tiab] OR "lesser developed nations"[tiab] OR "lesser developed population"[tiab] OR "lesser developed populations"[tiab] OR "lesser developed world"[tiab] OR "under developed country"[tiab] OR "under developed countries"[tiab] OR "under developed nation"[tiab] OR "under developed nations"[tiab] OR "under developed population"[tiab] OR "under developed populations"[tiab] OR "under developed world"[tiab] OR "underdeveloped country"[tiab] OR "underdeveloped countries"[tiab] OR "underdeveloped nation"[tiab] OR "underdeveloped nations"[tiab] OR "underdeveloped population"[tiab] OR "underdeveloped populations"[tiab] OR "underdeveloped world"[tiab] OR "middle income country"[tiab] OR "middle income countries"[tiab] OR "middle income nation"[tiab] OR "middle income nations"[tiab] OR "middle income population"[tiab] OR "middle income populations"[tiab] OR "low income country"[tiab] OR "low income countries"[tiab] OR "low income nation"[tiab] OR "low income nations"[tiab] OR "low income population"[tiab] OR "low income populations"[tiab] OR "lower income country"[tiab] OR "lower income countries"[tiab] OR "lower income nation"[tiab] OR "lower income nations"[tiab] OR "lower income population"[tiab] OR "lower income populations"[tiab] OR "underserved country"[tiab] OR "underserved countries"[tiab] OR "underserved nation"[tiab] OR "underserved nations"[tiab] OR "underserved population"[tiab] OR "underserved populations"[tiab] OR "underserved world"[tiab] OR "under served country"[tiab] OR "under served countries"[tiab] OR "under served nation"[tiab] OR "under served nations"[tiab] OR "under served population"[tiab] OR "under served populations"[tiab] OR "under served world"[tiab] OR "deprived country"[tiab] OR "deprived countries"[tiab] OR "deprived nation"[tiab] OR "deprived nations"[tiab] OR "deprived population"[tiab] OR "deprived populations"[tiab] OR "deprived world"[tiab] OR "poor country"[tiab] OR "poor countries"[tiab] OR "poor nation"[tiab] OR "poor nations"[tiab] OR "poor population"[tiab] OR "poor populations"[tiab] OR "poor world"[tiab] OR "poorer country"[tiab] OR "poorer countries"[tiab] OR "poorer nation"[tiab] OR "poorer nations"[tiab] OR "poorer population"[tiab] OR "poorer populations"[tiab] OR "poorer world"[tiab] OR "developing economy"[tiab] OR "developing economies"[tiab] OR "less developed economy"[tiab] OR "less developed economies"[tiab] OR "lesser developed economy"[tiab] OR "lesser developed economies"[tiab] OR "under developed economy"[tiab] OR "under developed economies"[tiab] OR "underdeveloped economy"[tiab] OR "underdeveloped economies"[tiab] OR "middle income economy"[tiab] OR "middle income economies"[tiab] OR "low income economy"[tiab] OR "low income economies"[tiab] OR "lower income economy"[tiab] OR "lower income economies"[tiab] OR "low gdp"[tiab] OR "low gnp"[tiab] OR "low gross domestic"[tiab] OR "low gross national"[tiab] OR "lower gdp"[tiab] OR "lower gnp"[tiab] OR "lower gross domestic"[tiab] OR "lower gross national"[tiab] OR lmic[tiab] OR lmics[tiab] OR "third world"[tiab] OR "lami country"[tiab] OR "lami countries"[tiab] OR "transitional country"[tiab] OR "transitional countries"[tiab] OR Africa[tiab] OR Asia[tiab] OR Caribbean[tiab] OR West Indies[tiab] OR South America[tiab] OR Latin America[tiab] OR Central America[tiab] OR "Atlantic Islands"[tiab] OR "Commonwealth of Independent States"[tiab] OR "Pacific Islands"[tiab] OR "Indian Ocean Islands"[tiab] OR "Eastern Europe"[tiab] OR Afghanistan[tiab] OR Albania[tiab] OR Algeria[tiab] OR Angola[tiab] OR Armenia[tiab] OR Armenian[tiab] OR Azerbaijan[tiab] OR Bangladesh[tiab] OR Benin[tiab] OR Byelarus[tiab] OR Byelorussian[tiab] OR Belarus[tiab] OR Belorussian[tiab] OR Belorussia[tiab] OR Belize[tiab] OR Bhutan[tiab] OR Bolivia[tiab] OR Bosnia[tiab] OR Herzegovina[tiab] OR Hercegovina[tiab] OR Botswana[tiab] OR Brasil[tiab] OR Brazil[tiab] OR Bulgaria[tiab] OR Burkina Faso[tiab] OR Burkina Fasso[tiab] OR Upper Volta[tiab] OR Burundi[tiab] OR Urundi[tiab] OR Cambodia[tiab] OR Khmer Republic[tiab] OR Kampuchea[tiab] OR Cameroon[tiab] OR Cameroons[tiab] OR Cameron[tiab] OR Cape Verde[tiab] OR Central African Republic[tiab] OR Chad[tiab] OR China[tiab] OR Colombia[tiab] OR Comoros[tiab] OR Comoro Islands[tiab] OR Comores[tiab] OR Mayotte[tiab] OR Congo[tiab] OR Zaire[tiab] OR Costa Rica[tiab] OR Cote d'Ivoire[tiab] OR Ivory Coast[tiab] OR Cuba[tiab] OR Czechoslovakia[tiab] OR Slovakia[tiab] OR Djibouti[tiab] OR French Somaliland[tiab] OR Dominica[tiab] OR Dominican Republic[tiab] OR East Timor[tiab] OR East Timur[tiab] OR Timor Leste[tiab] OR Ecuador[tiab] OR Egypt[tiab] OR El Salvador[tiab] OR Eritrea[tiab] OR Ethiopia[tiab] OR Fiji[tiab] OR Gabon[tiab] OR Gabonese Republic[tiab] OR Gambia[tiab] OR Gaza[tiab] OR Georgia Republic[tiab] OR Georgian Republic[tiab] OR Ghana[tiab] OR Gold Coast[tiab] OR Grenada[tiab] OR Guatemala[tiab] OR Guinea[tiab] OR Guiana[tiab] OR Guyana[tiab] OR Haiti[tiab] OR Honduras[tiab] OR India[tiab] OR Maldives[tiab] OR Indonesia[tiab] OR Iran[tiab] OR Iraq[tiab] OR Jamaica[tiab] OR Jordan[tiab] OR Kazakhstan[tiab] OR Kazakh[tiab] OR Kenya[tiab] OR Kiribati[tiab] OR Korea[tiab] OR Kosovo[tiab] OR Kyrgyzstan[tiab] OR Kirghizia[tiab] OR Kyrgyz Republic[tiab] OR Kirghiz[tiab] OR Kirgizstan[tiab] OR "Lao PDR"[tiab] OR Laos[tiab] OR Lebanon[tiab] OR Lesotho[tiab] OR Basutoland[tiab] OR Liberia[tiab] OR Libya[tiab] OR Macedonia[tiab] OR Madagascar[tiab] OR Malagasy Republic[tiab] OR Malaysia[tiab] OR Malaya[tiab] OR Malay[tiab] OR Sabah[tiab] OR Sarawak[tiab] OR Malawi[tiab] OR Nyasaland[tiab] OR Mali[tiab] OR Marshall Islands[tiab] OR Mauritania[tiab] OR Mauritius[tiab] OR Agalega Islands[tiab] OR "Melanesia"[tiab] OR Mexico[tiab] OR Micronesia[tiab] OR Middle East[tiab] OR Moldova[tiab] OR Moldovia[tiab] OR Moldovian[tiab] OR Mongolia[tiab] OR Montenegro[tiab] OR Morocco[tiab] OR Ifni[tiab] OR Mozambique[tiab] OR Myanmar[tiab] OR Myanma[tiab] OR Burma[tiab] OR Namibia[tiab] OR Nepal[tiab] OR Nicaragua[tiab] OR Niger[tiab] OR Nigeria[tiab] OR Muscat[tiab] OR Pakistan[tiab] OR Palau[tiab] OR Palestine[tiab] OR Panama[tiab] OR Paraguay[tiab] OR Peru[tiab] OR Philippines[tiab] OR Philipines[tiab] OR Phillipines[tiab] OR Phillippines[tiab] OR Romania[tiab] OR Rumania[tiab] OR Roumania[tiab] OR Rwanda[tiab] OR Ruanda[tiab] OR Saint Kitts[tiab] OR St Kitts[tiab] OR Nevis[tiab] OR Saint Lucia[tiab] OR St Lucia[tiab] OR Saint Vincent[tiab] OR St Vincent[tiab] OR Grenadines[tiab] OR Samoa[tiab] OR Samoan Islands[tiab] OR Navigator Island[tiab] OR Navigator Islands[tiab] OR Sao Tome[tiab] OR Senegal[tiab] OR Serbia[tiab] OR Montenegro[tiab] OR Sierra Leone[tiab] OR Sri Lanka[tiab] OR Ceylon[tiab] OR Solomon Islands[tiab] OR Somalia[tiab] OR Sudan[tiab] OR Suriname[tiab] OR Surinam[tiab] OR Swaziland[tiab] OR Syria[tiab] OR Syrian[tiab] OR Tajikistan[tiab] OR Tadzhikistan[tiab] OR Tadjikistan[tiab] OR Tadzhik[tiab] OR Tanzania[tiab] OR Thailand[tiab] OR Togo[tiab] OR Togolese Republic[tiab] OR Tonga[tiab] OR Tunisia[tiab] OR Turkey[tiab] OR Turkmenistan[tiab] OR Turkmen[tiab] OR Tuvalu[tiab] OR Uganda[tiab] OR Ukraine[tiab] OR Uzbekistan[tiab] OR Uzbek OR Vanuatu[tiab] OR New Hebrides[tiab] OR Vietnam[tiab] OR Viet Nam[tiab] OR West Bank[tiab] OR Yemen[tiab] OR Yugoslavia[tiab] OR Zambia[tiab] OR Zimbabwe[tiab] OR Rhodesia[tiab] OR Developing Countries[Mesh] OR Africa[Mesh:NoExp] OR Africa, Northern[Mesh:NoExp] OR Africa South of the Sahara[Mesh:NoExp] OR Africa, Central[Mesh:NoExp] OR Africa, Eastern[Mesh:NoExp] OR Africa, Southern[Mesh:NoExp] OR Africa, Western[Mesh:NoExp] OR Asia[Mesh:NoExp] OR Asia, Central[Mesh:NoExp] OR Asia, Southeastern[Mesh:NoExp] OR Asia, Western[Mesh:NoExp] OR Caribbean Region[Mesh:NoExp] OR West Indies[Mesh:NoExp] OR South America[Mesh:NoExp] OR Latin America[Mesh:NoExp] OR Central America[Mesh:NoExp] OR "Atlantic Islands"[Mesh:NoExp] OR "Commonwealth of Independent States"[Mesh:NoExp] OR "Pacific Islands"[Mesh:NoExp] OR "Indian Ocean Islands"[Mesh:NoExp] OR "Europe, Eastern"[Mesh:NoExp] OR Afghanistan[Mesh] OR Albania[Mesh] OR Algeria[Mesh] OR American Samoa[Mesh] OR Angola[Mesh] OR Armenia[Mesh] OR Azerbaijan[Mesh] OR "Baltic States"[Mesh] OR Bangladesh[Mesh] OR Benin[Mesh] OR "Republic of Belarus"[Mesh] OR Belize[Mesh] OR Bhutan[Mesh] OR Bolivia[Mesh] OR Bosnia-Herzegovina[Mesh] OR Botswana[Mesh] OR Brazil[Mesh] OR Bulgaria[Mesh] OR Burkina Faso[Mesh] OR Burundi[Mesh] OR Cambodia[Mesh] OR Cameroon[Mesh] OR Cape Verde[Mesh] OR Central African Republic[Mesh] OR Chad[Mesh]] OR China[Mesh] OR Colombia[Mesh] OR Comoros[Mesh] OR Congo[Mesh] OR Costa Rica[Mesh] OR Cote d'Ivoire[Mesh] OR Cuba[Mesh] OR Czechoslovakia[Mesh] OR Slovakia[Mesh] OR Djibouti[Mesh] OR "Democratic Republic of the Congo"[Mesh] OR "Democratic People's Republic of Korea"[Mesh] OR Dominica[Mesh] OR Dominican Republic[Mesh] OR East Timor[Mesh] OR Ecuador[Mesh] OR Egypt[Mesh] OR El Salvador[Mesh] OR Eritrea[Mesh] OR Ethiopia[Mesh] OR Fiji[Mesh] OR "French Guiana"[Mesh] OR Gabon[Mesh] OR Gambia[Mesh] OR "Georgia (Republic)"[Mesh] OR Ghana[Mesh] OR Grenada[Mesh] OR Guatemala[Mesh] OR Guinea[Mesh] OR Guinea-Bissau[Mesh] OR Guyana[Mesh] OR Haiti[Mesh] OR Honduras[Mesh] OR "Independent State of Samoa"[Mesh] OR India[Mesh] OR Indonesia[Mesh] OR Iran[Mesh] OR Iraq[Mesh] OR Jamaica[Mesh] OR Jordan[Mesh] OR Kazakhstan[Mesh] OR Kenya[Mesh] OR Korea[Mesh] OR Kyrgyzstan[Mesh] OR Laos[Mesh] OR Lebanon[Mesh] OR Lesotho[Mesh] OR Liberia[Mesh] OR Libya[Mesh] OR "Macedonia (Republic)"[Mesh] OR Madagascar[Mesh] OR Malawi[Mesh] OR Malaysia[Mesh] OR Mali[Mesh] OR Mauritania[Mesh] OR Mauritius[Mesh] OR "Melanesia"[Mesh] OR Mexico[Mesh] OR Micronesia[Mesh] OR Middle East[Mesh:NoExp] OR Moldova[Mesh] OR Mongolia[Mesh] OR Montenegro[Mesh] OR Morocco[Mesh] OR Mozambique[Mesh] OR Myanmar[Mesh] OR Namibia[Mesh] OR Nepal[Mesh] OR Nicaragua[Mesh] OR Niger[Mesh] OR Nigeria[Mesh] OR Pakistan[Mesh] OR Palau[Mesh] OR Panama[Mesh] OR Papua New Guinea[Mesh] OR Paraguay[Mesh] OR Peru[Mesh] OR Philippines[Mesh] OR "Republic of Korea"[Mesh] OR Romania[Mesh] OR Rwanda[Mesh] OR Saint Lucia[Mesh] OR "Saint Vincent and the Grenadines"[Mesh] OR Samoa[Mesh] OR Senegal[Mesh] OR Serbia[Mesh] OR Montenegro[Mesh] OR Sierra Leone[Mesh] OR Sri Lanka[Mesh] OR Somalia[Mesh] OR South Africa[Mesh] OR Sudan[Mesh] OR Suriname[Mesh] OR Swaziland[Mesh] OR Syria[Mesh] OR Tajikistan[Mesh] OR Tanzania[Mesh] OR Thailand[Mesh] OR Togo[Mesh] OR Tonga[Mesh] OR Tunisia[Mesh] OR Turkey[Mesh] OR Turkmenistan[Mesh] OR Uganda[Mesh] OR Ukraine[Mesh] OR Uzbekistan[Mesh] OR Vanuatu[Mesh] OR Vietnam[Mesh] OR Yemen[Mesh] OR Yugoslavia[Mesh] OR Zambia[Mesh] OR Zimbabwe[Mesh] OR "Southern African Development Community"[tw] OR "East African Community"[tw] OR "West African Health Organisation"[tw] OR "Sub Saharan Africa "[tw] OR "SubSaharan Africa "[tw])

**Concept 5: Animals**

("animals"[MeSH Terms] NOT ("humans"[MeSH Terms] AND "animals"[MeSH Terms]))

**EconLit**

**Concept 1: Disease**

"Buruli Ulcer" OR "Bairnsdale ulcer" OR "Buruli disease" OR " Mycobacterium ulcerans Infection" OR "Mycobacterium Infections" OR "Mycobacterium Infection" OR "mycobacteriosis" OR "myobacteriosis” OR "Chagas" OR "Trypanosomiasis" OR "Trypanosoma cruzi" OR "Cholera" OR "cholera" OR "Choleras" OR "Chikungunya" OR "chickungunya" OR "Crimean Hemorrhagic Fever" OR "Congo Virus Infections" OR "Crimean Hemorrhagic Fevers" OR "Congo Hemorrhagic Fevers" OR "Congo Virus Infection" OR "Dengue" OR "Breakbone Fever" OR "Break bone Fever" OR "dandy fever" OR "red fever" OR "solar fever" OR "diarrhea" OR "diarrhoea" OR "diarrheal" OR "diarrhoeal" OR "dysentery" OR "Gastrointestinal Disease" OR "gastrointestinal diseases" OR "Diphtheria" OR "Diphtherias" OR diphteria OR diphtheriae OR "Dracunculiases" OR "Guinea Worm Infection" OR "Guinea Worm Infections" OR "Guinea Worm Disease" OR "Guinea Worm Diseases" OR "Dracunculosis" OR "Dracunculoses" OR "ebola" OR "Echinococcosis" OR "Echinococcoses" OR "Hydatid Cyst" OR "Hydatid Cysts" OR "Hydatid disease" OR "Hydatidosis" OR "Hydatidoses" OR "hydatid lung cyst" OR "lung echinococcal cyst" OR "lung echinococcosis" OR "lung echinococcus" OR "lung hydatid disease" OR "lung hydatidosis" OR "pulmonary echinococcal cyst" OR "pulmonary echinococcosis" OR "pulmonary Echinococcus cyst" OR "pulmonary hydatid cyst" OR "pulmonary hydatidosis" OR "Foodborne trematodiases" OR "Gastroenteritis" OR Gastroenteritide OR "gastro enteritis" OR gastroduodenitis OR "gastrointestinal acute infection" OR "type a hepatitis" OR "Hepatitis type A" OR "Hepatitis A" OR "hep A" OR "botkin disease" OR "Hepatovirus" OR "type b Hepatitis" OR "Hepatitis type B" OR "Hepatitis B" OR "non a non b hepatitis" OR "Hepatitis E" OR "Hepatitides" OR "ET-NANBH" OR "Acquired Immunodeficiency Syndrome" OR "human immunodeficiency virus" OR "human immuno deficiency virus" OR "immuno deficiency associated virus" OR "immunodeficiency associated virus" OR "human immune deficiency virus" OR HIV OR AIDS OR "Papillomaviridae" OR "Human Papilloma Virus" OR "Human Papilloma Viruses" OR "Human Papillomavirus" OR ((human OR humans) AND (Influenzas OR influenza OR flu)) OR "Japanese B Viral Encephalitis" OR "Japanese B Encephalitis" OR "Japanese Encephalitis" OR "Lassa Fever" OR "Lassa Fevers" OR "Leishmaniasis" OR "black fever" OR "leishmaniosis" OR "kala azar" OR "lymphatic filariasis" OR "filarial lymphedema" OR "filarial lymphooedema" OR "elephantiasis" OR "Leprosy" OR "Leprosies" OR "Hansen Disease" OR "Hansens Disease" OR "Lymphatic filariasis" OR "Filarial Elephantiases" OR "Filarial Elephantiasis" OR "Lymphatic Filariases" OR "Bancroftian Elephantiases" OR "Malaria" OR "paludism" OR "Marburg Virus Disease" OR "Marburg Disease" OR "Marburg Hemorrhagic Fever" OR "Marburg syndrome" OR "Marburg disease" OR "Marburg virus disease" OR "Meningococcal" OR meningococcaemia OR "meningococcic" OR meningococceal OR "meningococci" OR "Meningococcus" OR "Micrococcus intracellularis" OR "Neisseria weichselbaumi" OR "meningitides" OR "Meningitis" OR "Rubella" OR "Rubellas" OR "epidemic roseola" OR "Monkeypox" OR "Monkey pox" OR "Mumps" OR "Parotitis" OR "parodotis" OR "parotid inflammation" OR partitis OR Onchocerciases OR "Onchocerca infection" OR "Onchocerca volvulus infection" OR "Onchocerca infections" OR "Onchocerca volvulus infections" OR "onchocercosis" OR "onchocerosis" OR "Whooping Cough" OR "Pertussis" OR "Pertusses" OR "Plague" OR "Yersinia pestis" OR "parotiditis" OR "Pneumococcal infections" OR "Pneumococcal infection" OR "pneumococci infection" OR "pneumococci infections" OR "pneumococcal disease" OR pneumococcosis OR "pneumococcus infection" OR "pneumococcus infections" OR "pneumoniae Infections" OR "pneumoniae Infection" OR "Streptococcus pneumonia" OR "S.pneumoniae" OR "Poliomyelitis" OR "polio" or "poliovirus" OR "polios" OR "Infantile Paralysis" OR "Hubert disease" OR "lyssavirus" OR "lytta" OR "Hydrophobia" OR "Lyssa" OR "Lyssas" OR "Rift Valley Fever" OR "Measles" OR "Rubeola" OR "morbilli" OR "Smallpox" OR "Small pox" OR "Variola" OR "Variolas" OR "Alastrim" OR "Helminthiasis" OR "Helminthiases" OR "Nematomorpha Infection" OR "Nematode Infections" OR "Nematode Infection" OR "Nematomorpha Infections" OR "soil transmitted Helminth infection" OR "soil transmitted Helminth infections" OR "Larva migrans syndrome" OR (("roundworm" OR "whipworm" OR "Ascaris lumbricoides" OR "Ascaris lumbricoide" OR "Trichuris trichiura" OR "hookworms" OR "hookworm" OR "Necator americanus" OR "Ancylostoma duodenale" OR "Enterobius vermicularis" OR Taenia OR Secernentea OR helminth OR helminthic OR helminthosis OR worm or trypanosomiasis OR Trypanosoma) AND ("infections" OR "infection" OR infestation OR infestations)) OR "Taeniasis" OR "Taeniases" OR "Cysticercosis" OR Cysticercoses OR "Tetanus" OR "Trachoma" OR Trachomas OR "Chlamydia conjunctivitis" OR "Chlamydia trachomatis conjunctivitis" OR "chlamydial conjunctivitis" OR "granular conjunctivitis" OR "Egyptian Ophthalmia" OR "Trypanosomiasis" OR "Trypanosomiases" OR "Trypanosoma infection" OR "trypanosome infection" OR "Trypanosoma infections" OR "trypanosome infections" OR trypanosomosis OR "African Trypanosomiases" OR "African Trypanosomiasis" OR "African Sleeping Sickness" OR "African Sleeping Sicknesses" OR "Nagana" OR "Tuberculosis" OR "Tuberculoses" OR "Kochs Disease" OR "Koch Disease" OR "Human Herpesvirus 3" OR "Chickenpox" OR "Chicken pox" OR "Herpes zoster" OR "HHV-3" OR "Herpesvirus 3" OR "VZ Virus" OR "VZ Viruses" OR "Herpesvirus Varicellae" OR "varicella" OR "Yaws" OR Frambesia OR Frambesias OR framboesia OR parangi OR pian OR "Treponema pertenue infection" OR "Yellow Fever" OR "Yellow Fevers" OR "Zika Virus"

**Concept 2: Children**

"infant" OR "infants" OR "neonate" OR "neonates" OR "neonatal" OR "newborn" OR "newborns" OR "new-born" OR "new-borns" OR "baby" OR "babies" OR "Premature" OR "preterm" OR "pre term" OR "child" OR "children" OR "youth" OR "youths" OR "young people" OR "childhood" OR "toddler" OR "toddlers" OR "kid" OR "kids" OR "young patient" OR "young patients" OR "boy" OR "boys" OR "girl" OR "girls" OR "young age" OR "pediatric" OR "pre-schooler" OR "preschooler" OR "under 5" OR "under five" OR "under fives" OR "less than five" OR "Adolescents" OR "Adolescence" OR "Teens" OR "Teen" OR "Teenagers" OR "Teenager" OR "Youth" OR "Youths" OR "young adult" OR "young adults" OR "Minor" OR "Minors"

**Concept 3: Cost**

"Economics" OR "Economic" OR "pricing" OR cost OR Costs OR "Burden of Illness" OR "Illness Burden" OR "Illness Burdens" OR "value of life" OR "financial" OR "finance" OR "financing" OR "price" OR "prices" OR "market" OR "spending" OR "expenditure"

**Concept 4: LMIC**

("developing country" OR "developing countries" OR "developing nation" OR "developing nations" OR "developing population" OR "developing populations" OR "developing world" OR "less developed country" OR "less developed countries" OR "less developed nation" OR "less developed nations" OR "less developed population" OR "less developed populations" OR "less developed world" OR "lesser developed country" OR "lesser developed countries" OR "lesser developed nation" OR "lesser developed nations" OR "lesser developed population" OR "lesser developed populations" OR "lesser developed world" OR "under developed country" OR "under developed countries" OR "under developed nation" OR "under developed nations" OR "under developed population" OR "under developed populations" OR "under developed world" OR "underdeveloped country" OR "underdeveloped countries" OR "underdeveloped nation" OR "underdeveloped nations" OR "underdeveloped population" OR "underdeveloped populations" OR "underdeveloped world" OR "middle income country" OR "middle income countries" OR "middle income nation" OR "middle income nations" OR "middle income population" OR "middle income populations" OR "low income country" OR "low income countries" OR "low income nation" OR "low income nations" OR "low income population" OR "low income populations" OR "lower income country" OR "lower income countries" OR "lower income nation" OR "lower income nations" OR "lower income population" OR "lower income populations" OR "underserved country" OR "underserved countries" OR "underserved nation" OR "underserved nations" OR "underserved population" OR "underserved populations" OR "underserved world" OR "under served country" OR "under served countries" OR "under served nation" OR "under served nations" OR "under served population" OR "under served populations" OR "under served world" OR "deprived country" OR "deprived countries" OR "deprived nation" OR "deprived nations" OR "deprived population" OR "deprived populations" OR "deprived world" OR "poor country" OR "poor countries" OR "poor nation" OR "poor nations" OR "poor population" OR "poor populations" OR "poor world" OR "poorer country" OR "poorer countries" OR "poorer nation" OR "poorer nations" OR "poorer population" OR "poorer populations" OR "poorer world" OR "developing economy" OR "developing economies" OR "less developed economy" OR "less developed economies" OR "lesser developed economy" OR "lesser developed economies" OR "under developed economy" OR "under developed economies" OR "underdeveloped economy" OR "underdeveloped economies" OR "middle income economy" OR "middle income economies" OR "low income economy" OR "low income economies" OR "lower income economy" OR "lower income economies" OR "low gdp" OR "low gnp" OR "low gross domestic" OR "low gross national" OR "lower gdp" OR "lower gnp" OR "lower gross domestic" OR "lower gross national" OR "lmic" OR "lmics" OR "third world" OR "lami country" OR "lami countries" OR "transitional country" OR "transitional countries" OR "Africa" OR "Asia" OR "Caribbean" OR "West Indies" OR "South America" OR "Latin America" OR "Central America" OR "Atlantic Islands" OR "Commonwealth of Independent States" OR "Pacific Islands" OR "Indian Ocean Islands" OR "Eastern Europe" OR "Afghanistan" OR "Albania" OR "Algeria" OR "Angola" OR "Antigua" OR "Barbuda" OR "Argentina" OR "Armenia" OR "Armenian" OR "Aruba" OR "Azerbaijan" OR "Bahrain" OR "Bangladesh" OR "Barbados" OR "Benin" OR "Byelarus" OR "Byelorussian" OR "Belarus" OR "Belorussian" OR "Belorussia" OR "Belize" OR "Bhutan" OR "Bolivia" OR "Bosnia" OR "Herzegovina" OR "Hercegovina" OR "Botswana" OR "Brasil" OR "Brazil" OR "Bulgaria" OR "Burkina Faso" OR "Burkina Fasso" OR "Upper Volta" OR "Burundi" OR "Urundi" OR "Cambodia" OR "Khmer Republic" OR "Kampuchea" OR "Cameroon" OR "Cameroons" OR "Cameron" OR "Camerons" OR "Cape Verde" OR "Central African Republic" OR "Chad" OR "Chile" OR "China" OR "Colombia" OR "Comoros" OR "Comoro Islands" OR "Comores" OR "Mayotte" OR "Congo" OR "Zaire" OR "Costa Rica" OR "Cote d'Ivoire" OR "Ivory Coast" OR "Croatia" OR "Cuba" OR "Cyprus" OR "Czechoslovakia" OR "Czech Republic" OR "Slovakia" OR "Slovak Republic" OR "Djibouti" OR "French Somaliland" OR "Dominica" OR "Dominican Republic" OR "East Timor" OR "East Timur" OR "Timor Leste" OR "Ecuador" OR "Egypt" OR "United Arab Republic" OR "El Salvador" OR "Eritrea" OR "Estonia" OR "Ethiopia" OR "Fiji" OR "Gabon" OR "Gabonese Republic" OR "Gambia" OR "Gaza" OR "Georgia Republic" OR "Georgian Republic" OR "Ghana" OR "Gold Coast" OR "Greece" OR "Grenada" OR "Guatemala" OR "Guinea" OR "Guam" OR "Guiana" OR "Guyana" OR "Haiti" OR "Honduras" OR "Hungary" OR "India" OR "Maldives" OR "Indonesia" OR "Iran" OR "Iraq" OR "Isle of Man" OR "Jamaica" OR "Jordan" OR "Kazakhstan" OR "Kazakh" OR "Kenya" OR "Kiribati" OR "Korea" OR "Kosovo" OR "Kyrgyzstan" OR "Kirghizia" OR "Kyrgyz Republic" OR "Kirghiz" OR "Kirgizstan" OR "Lao PDR" OR "Laos" OR "Latvia" OR "Lebanon" OR "Lesotho" OR "Basutoland" OR "Liberia" OR "Libya" OR "Lithuania" OR "Macedonia" OR "Madagascar" OR "Malagasy Republic" OR "Malaysia" OR "Malaya" OR "Malay" OR "Sabah" OR "Sarawak" OR "Malawi" OR "Nyasaland" OR "Mali" OR "Malta" OR "Marshall Islands" OR "Mauritania" OR "Mauritius" OR "Agalega Islands" OR "Melanesia" OR "Mexico" OR "Micronesia" OR "Middle East" OR "Moldova" OR "Moldovia" OR "Moldovian" OR "Mongolia" OR "Montenegro" OR "Morocco" OR "Ifni" OR "Mozambique" OR "Myanmar" OR "Myanma" OR "Burma" OR "Namibia" OR "Nepal" OR "Netherlands Antilles" OR "New Caledonia" OR "Nicaragua" OR "Niger" OR "Nigeria" OR "Northern Mariana Islands" OR "Oman" OR "Muscat" OR "Pakistan" OR "Palau" OR "Palestine" OR "Panama" OR "Paraguay" OR "Peru" OR "Philippines" OR "Philipines" OR "Phillipines" OR "Phillippines" OR "Poland" OR "Portugal" OR "Puerto Rico" OR "Romania" OR "Rumania" OR "Roumania" OR "Russia" OR "Russian" OR "Rwanda" OR "Ruanda" OR "Saint Kitts" OR "St Kitts" OR "Nevis" OR "Saint Lucia" OR "St Lucia" OR "Saint Vincent" OR "St Vincent" OR "Grenadines" OR "Samoa" OR "Samoan Islands" OR "Navigator Island" OR "Navigator Islands" OR "Sao Tome" OR "Saudi Arabia" OR "Senegal" OR "Serbia" OR "Montenegro" OR "Seychelles" OR "Sierra Leone" OR "Slovenia" OR "Sri Lanka" OR "Ceylon" OR "Solomon Islands" OR "Somalia" OR "Sudan" OR "Suriname" OR "Surinam" OR "Swaziland" OR "Syria" OR "Syrian" OR "Tajikistan" OR "Tadzhikistan" OR "Tadjikistan" OR "Tadzhik" OR "Tanzania" OR "Thailand" OR "Togo" OR "Togolese Republic" OR "Tonga" OR "Trinidad" OR "Tobago" OR "Tunisia" OR "Turkey" OR "Turkmenistan" OR "Turkmen" OR "Tuvalu" OR "Uganda" OR "Ukraine" OR "Uruguay" OR "USSR" OR "Soviet Union" OR "Union of Soviet Socialist Republics" OR "Uzbekistan" OR "Uzbek" OR "Vanuatu" OR "New Hebrides" OR "Venezuela" OR "Vietnam" OR "Viet Nam" OR "West Bank" OR "Yemen" OR "Yugoslavia" OR "Zambia" OR "Zimbabwe" OR "Rhodesia" OR "Caribbean Region" OR "Central America" OR "Baltic States" OR "Republic of Belarus" OR "French Guiana" OR "Independent State of Samoa")

**Concept 5: Animals**

("animals"[MeSH Terms] NOT ("humans"[MeSH Terms] AND "animals"[MeSH Terms]))

**Embase**

**Concept 1: Disease**

'Buruli ulcer'/exp OR 'mycobacteriosis'/exp OR "Chagas Disease"/exp OR "Cholera"/exp OR "Chikungunya"/exp OR "Chikungunya virus"/exp OR 'Crimean Congo hemorrhagic fever'/exp OR "Dengue"/exp OR "Severe Dengue"/exp OR "diarrhea"/exp OR "dysentery"/exp OR "gastrointestinal diseases"/exp OR "Diphtheria"/exp OR 'Ebola hemorrhagic fever'/exp OR 'echinococcosis'/exp OR "Ebolavirus"/exp OR "Gastroenteritis"/exp OR "Hepatitis A"/exp OR "Hepatitis B"/exp OR 'chronic hepatitis B'/exp OR 'chronic hepatitis c'/exp OR "Hepatitis C"/exp OR 'hepatitis E'/exp OR 'liver hydatid cyst'/exp OR 'lung hydatid cyst'/exp OR 'Human immunodeficiency virus'/exp OR 'Papillomaviridae'/exp OR "Influenza"/exp OR 'Japanese encephalitis'/exp OR "Lassa Fever"/exp OR "Leishmaniasis"/exp OR 'lymphatic filariasis'/exp OR "Malaria"/exp OR 'Marburg hemorrhagic fever'/exp OR "Neisseria meningitides"/exp OR 'meningococcosis'/exp OR 'epidemic meningitis'/exp OR "Measles"/exp OR "Rubella"/exp OR "Monkeypox"/exp OR "Mumps"/exp OR "Onchocerciasis"/exp OR "Whooping Cough"/exp OR "Plague"/exp OR "Pneumococcal Infection"/exp OR 'pneumococcal meningitis'/exp OR "Pneumonia"/exp OR "Poliomyelitis"/exp OR "Rabies"/exp OR "Rabies virus"/exp OR "Rift Valley Fever"/exp OR "Rubella"/exp OR "Smallpox"/exp OR 'nematodiasis'/exp OR "Taeniasis"/exp OR "Cysticercosis"/exp OR "Tetanus"/exp OR "Trachoma"/exp OR "Trypanosomiasis"/exp OR "Tuberculosis"/exp OR "Helminthiasis"/exp OR "Ascaris lumbricoides"/exp OR 'African trypanosomiasis'/exp OR 'Varicella zoster virus'/exp OR "Chickenpox"/exp OR "Yaws"/exp OR "Yellow Fever"/exp OR "Yellow fever virus"/exp OR "Zika fever"/exp OR "Zika Virus"/exp OR "Leprosy"/exp OR ("Buruli Ulcer" OR "Bairnsdale ulcer" OR "Buruli disease" OR " Mycobacterium ulcerans Infection" OR "Mycobacterium Infections" OR "Mycobacterium Infection" OR "mycobacteriosis" OR "myobacteriosis” OR "Chagas" OR "Trypanosomiasis" OR "Trypanosoma cruzi" OR "Cholera" OR "cholera" OR "Choleras" OR "Chikungunya" OR "chickungunya" OR "Crimean Hemorrhagic Fever" OR "Congo Virus Infections" OR "Crimean Hemorrhagic Fevers" OR "Congo Hemorrhagic Fevers" OR "Congo Virus Infection" OR "Dengue" OR "Breakbone Fever" OR "Break bone Fever" OR "dandy fever" OR "red fever" OR "solar fever" OR "diarrhea" OR "diarrhoea" OR "diarrheal" OR "diarrhoeal" OR "dysentery" OR "Gastrointestinal Disease" OR "gastrointestinal diseases" OR "Diphtheria" OR "Diphtherias" OR diphteria OR diphtheriae OR "Dracunculiases" OR "Guinea Worm Infection" OR "Guinea Worm Infections" OR "Guinea Worm Disease" OR "Guinea Worm Diseases" OR "Dracunculosis" OR "Dracunculoses" OR "ebola" OR "Echinococcosis" OR "Echinococcoses" OR "Hydatid Cyst" OR "Hydatid Cysts" OR "Hydatid disease" OR "Hydatidosis" OR "Hydatidoses" OR "hydatid lung cyst" OR "lung echinococcal cyst" OR "lung echinococcosis" OR "lung echinococcus" OR "lung hydatid disease" OR "lung hydatidosis" OR "pulmonary echinococcal cyst" OR "pulmonary echinococcosis" OR "pulmonary Echinococcus cyst" OR "pulmonary hydatid cyst" OR "pulmonary hydatidosis" OR "Foodborne trematodiases" OR "Gastroenteritis" OR Gastroenteritide OR "gastro enteritis" OR gastroduodenitis OR "gastrointestinal acute infection" OR "type a hepatitis" OR "Hepatitis type A" OR "Hepatitis A" OR "hep A" OR "botkin disease" OR "Hepatovirus" OR "type b Hepatitis" OR "Hepatitis type B" OR "Hepatitis B" OR "non a non b hepatitis" OR "Hepatitis E" OR "Hepatitides" OR "ET-NANBH" OR "Acquired Immunodeficiency Syndrome" OR "human immunodeficiency virus" OR "human immuno deficiency virus" OR "immuno deficiency associated virus" OR "immunodeficiency associated virus" OR "human immune deficiency virus" OR HIV OR AIDS OR "Papillomaviridae" OR "Human Papilloma Virus" OR "Human Papilloma Viruses" OR "Human Papillomavirus" OR ((human OR humans) AND (Influenzas OR influenza OR flu)) OR "Japanese B Viral Encephalitis" OR "Japanese B Encephalitis" OR "Japanese Encephalitis" OR "Lassa Fever" OR "Lassa Fevers" OR "Leishmaniasis" OR "black fever" OR "leishmaniosis" OR "kala azar" OR "lymphatic filariasis" OR "filarial lymphedema" OR "filarial lymphooedema" OR "elephantiasis" OR "Leprosy" OR "Leprosies" OR "Hansen Disease" OR "Hansens Disease" OR "Lymphatic filariasis" OR "Filarial Elephantiases" OR "Filarial Elephantiasis" OR "Lymphatic Filariases" OR "Bancroftian Elephantiases" OR "Malaria" OR "paludism" OR "Marburg Virus Disease" OR "Marburg Disease" OR "Marburg Hemorrhagic Fever" OR "Marburg syndrome" OR "Marburg disease" OR "Marburg virus disease" OR "Meningococcal" OR meningococcaemia OR "meningococcic" OR meningococceal OR "meningococci" OR "Meningococcus" OR "Micrococcus intracellularis" OR "Neisseria weichselbaumi" OR "meningitides" OR "Meningitis" OR "Rubella" OR "Rubellas" OR "epidemic roseola" OR "Monkeypox" OR "Monkey pox" OR "Mumps" OR "Parotitis" OR "parodotis" OR "parotid inflammation" OR partitis OR Onchocerciases OR "Onchocerca infection" OR "Onchocerca volvulus infection" OR "Onchocerca infections" OR "Onchocerca volvulus infections" OR "onchocercosis" OR "onchocerosis" OR "Whooping Cough" OR "Pertussis" OR "Pertusses" OR "Plague" OR "Yersinia pestis" OR "parotiditis" OR "Pneumococcal infections" OR "Pneumococcal infection" OR "pneumococci infection" OR "pneumococci infections" OR "pneumococcal disease" OR pneumococcosis OR "pneumococcus infection" OR "pneumococcus infections" OR "pneumoniae Infections" OR "pneumoniae Infection" OR "Streptococcus pneumonia" OR "S.pneumoniae" OR "Poliomyelitis" OR "polio" or "poliovirus" OR "polios" OR "Infantile Paralysis" OR "Hubert disease" OR "lyssavirus" OR "lytta" OR "Hydrophobia" OR "Lyssa" OR "Lyssas" OR "Rift Valley Fever" OR "Measles" OR "Rubeola" OR "morbilli" OR "Smallpox" OR "Small pox" OR "Variola" OR "Variolas" OR "Alastrim" OR "Helminthiasis" OR "Helminthiases" OR "Nematomorpha Infection" OR "Nematode Infections" OR "Nematode Infection" OR "Nematomorpha Infections" OR "soil transmitted Helminth infection" OR "soil transmitted Helminth infections" OR "Larva migrans syndrome" OR (("roundworm" OR "whipworm" OR "Ascaris lumbricoides" OR "Ascaris lumbricoide" OR "Trichuris trichiura" OR "hookworms" OR "hookworm" OR "Necator americanus" OR "Ancylostoma duodenale" OR "Enterobius vermicularis" OR Taenia OR Secernentea OR helminth OR helminthic OR helminthosis OR worm or trypanosomiasis OR Trypanosoma) AND ("infections" OR "infection" OR infestation OR infestations)) OR "Taeniasis" OR "Taeniases" OR "Cysticercosis" OR Cysticercoses OR "Tetanus" OR "Trachoma" OR Trachomas OR "Chlamydia conjunctivitis" OR "Chlamydia trachomatis conjunctivitis" OR "chlamydial conjunctivitis" OR "granular conjunctivitis" OR "Egyptian Ophthalmia" OR "Trypanosomiasis" OR "Trypanosomiases" OR "Trypanosoma infection" OR "trypanosome infection" OR "Trypanosoma infections" OR "trypanosome infections" OR trypanosomosis OR "African Trypanosomiases" OR "African Trypanosomiasis" OR "African Sleeping Sickness" OR "African Sleeping Sicknesses" OR "Nagana" OR "Tuberculosis" OR "Tuberculoses" OR "Kochs Disease" OR "Koch Disease" OR "Human Herpesvirus 3" OR "Chickenpox" OR "Chicken pox" OR "Herpes zoster" OR "HHV-3" OR "Herpesvirus 3" OR "VZ Virus" OR "VZ Viruses" OR "Herpesvirus Varicellae" OR "varicella" OR "Yaws" OR Frambesia OR Frambesias OR framboesia OR parangi OR pian OR "Treponema pertenue infection" OR "Yellow Fever" OR "Yellow Fevers" OR "Zika Virus"):ab,ti

**Concept 2: Children**

"Infant"/exp OR "Newborn"/exp OR "child"/exp OR "Preschool child"/exp OR 'minor (person)'/exp OR "Adolescent"/exp OR "Young Adult"/exp OR ("infant" OR "infants" OR "neonate" OR "neonates" OR "neonatal" OR "newborn" OR "newborns" OR "new-born" OR "new-borns" OR "baby" OR "babies" OR "Premature" OR "preterm" OR "pre term" OR "child" OR "children" OR "youth" OR "youths" OR "young people" OR "childhood" OR "toddler" OR "toddlers" OR "kid" OR "kids" OR "young patient" OR "young patients" OR "boy" OR "boys" OR "girl" OR "girls" OR "young age" OR "pediatric" OR "pre-schooler" OR "preschooler" OR "under 5" OR "under five" OR "under fives" OR "less than five" OR "Adolescents" OR "Adolescence" OR "Teens" OR "Teen" OR "Teenagers" OR "Teenager" OR "Youth" OR "Youths" OR "young adult" OR "young adults" OR "Minor" OR "Minors"):ab,ti

**Concept 3: Cost**

"Economics"/exp OR "cost"/exp OR "cost-benefit analysis"/exp OR "cost control"/exp OR "cost of illness"/exp OR "health care cost"/exp OR ("Economics" OR "Economic" OR "pricing" OR cost OR Costs OR "Burden of Illness" OR "Illness Burden" OR "Illness Burdens" OR "value of life" OR "financial" OR "finance" OR "financing" OR "price" OR "prices" OR "market" OR "spending" OR "expenditure"):ab,ti

**Concept 4: LMIC**

'developing country':ab,ti OR 'developing countries':ab,ti OR 'developing nation':ab,ti OR 'developing nations':ab,ti OR 'developing population':ab,ti OR 'developing populations':ab,ti OR 'developing world':ab,ti OR 'less developed country':ab,ti OR 'less developed countries':ab,ti OR 'less developed nation':ab,ti OR 'less developed nations':ab,ti OR 'less developed population':ab,ti OR 'less developed populations':ab,ti OR 'less developed world':ab,ti OR 'lesser developed country':ab,ti OR 'lesser developed countries':ab,ti OR 'lesser developed nation':ab,ti OR 'lesser developed nations':ab,ti OR 'lesser developed population':ab,ti OR 'lesser developed populations':ab,ti OR 'lesser developed world':ab,ti OR 'under developed country':ab,ti OR 'under developed countries':ab,ti OR 'under developed nation':ab,ti OR 'under developed nations':ab,ti OR 'under developed population':ab,ti OR 'under developed populations':ab,ti OR 'under developed world':ab,ti OR 'underdeveloped country':ab,ti OR 'underdeveloped countries':ab,ti OR 'underdeveloped nation':ab,ti OR 'underdeveloped nations':ab,ti OR 'underdeveloped population':ab,ti OR 'underdeveloped populations':ab,ti OR 'underdeveloped world':ab,ti OR 'middle income country':ab,ti OR 'middle income countries':ab,ti OR 'middle income nation':ab,ti OR 'middle income nations':ab,ti OR 'middle income population':ab,ti OR 'middle income populations':ab,ti OR 'low income country':ab,ti OR 'low income countries':ab,ti OR 'low income nation':ab,ti OR 'low income nations':ab,ti OR 'low income population':ab,ti OR 'low income populations':ab,ti OR 'lower income country':ab,ti OR 'lower income countries':ab,ti OR 'lower income nation':ab,ti OR 'lower income nations':ab,ti OR 'lower income population':ab,ti OR 'lower income populations':ab,ti OR 'underserved country':ab,ti OR 'underserved countries':ab,ti OR 'underserved nation':ab,ti OR 'underserved nations':ab,ti OR 'underserved population':ab,ti OR 'underserved populations':ab,ti OR 'underserved world':ab,ti OR 'under served country':ab,ti OR 'under served countries':ab,ti OR 'under served nation':ab,ti OR 'under served nations':ab,ti OR 'under served population':ab,ti OR 'under served populations':ab,ti OR 'under served world':ab,ti OR 'deprived country':ab,ti OR 'deprived countries':ab,ti OR 'deprived nation':ab,ti OR 'deprived nations':ab,ti OR 'deprived population':ab,ti OR 'deprived populations':ab,ti OR 'deprived world':ab,ti OR 'poor country':ab,ti OR 'poor countries':ab,ti OR 'poor nation':ab,ti OR 'poor nations':ab,ti OR 'poor population':ab,ti OR 'poor populations':ab,ti OR 'poor world':ab,ti OR 'poorer country':ab,ti OR 'poorer countries':ab,ti OR 'poorer nation':ab,ti OR 'poorer nations':ab,ti OR 'poorer population':ab,ti OR 'poorer populations':ab,ti OR 'poorer world':ab,ti OR 'developing economy':ab,ti OR 'developing economies':ab,ti OR 'less developed economy':ab,ti OR 'less developed economies':ab,ti OR 'lesser developed economy':ab,ti OR 'lesser developed economies':ab,ti OR 'under developed economy':ab,ti OR 'under developed economies':ab,ti OR 'underdeveloped economy':ab,ti OR 'underdeveloped economies':ab,ti OR 'middle income economy':ab,ti OR 'middle income economies':ab,ti OR 'low income economy':ab,ti OR 'low income economies':ab,ti OR 'lower income economy':ab,ti OR 'lower income economies':ab,ti OR 'low gdp':ab,ti OR 'low gnp':ab,ti OR 'low gross domestic':ab,ti OR 'low gross national':ab,ti OR 'lower gdp':ab,ti OR 'lower gnp':ab,ti OR 'lower gross domestic':ab,ti OR 'lower gross national':ab,ti OR lmic:ab,ti OR lmics:ab,ti OR 'third world':ab,ti OR 'lami country':ab,ti OR 'lami countries':ab,ti OR 'transitional country':ab,ti OR 'transitional countries':ab,ti OR Africa:ti,ab OR Asia:ti,ab OR Caribbean:ti,ab OR 'West Indies':ti,ab OR 'South America':ti,ab OR 'Latin America':ti,ab OR 'Central America':ti,ab OR 'atlantic islands':ab,ti OR 'commonwealth of independent states':ab,ti OR 'pacific islands':ab,ti OR 'indian ocean islands':ab,ti OR 'eastern europe':ab,ti OR Afghanistan:ti,ab OR Albania:ti,ab OR Algeria:ti,ab OR Angola:ti,ab OR Antigua:ti,ab OR Barbuda:ti,ab OR Argentina:ti,ab OR Armenia:ti,ab OR Armenian:ti,ab OR Aruba:ti,ab OR Azerbaijan:ti,ab OR Bahrain:ti,ab OR Bangladesh:ti,ab OR Barbados:ti,ab OR Benin:ti,ab OR Byelarus:ti,ab OR Byelorussian:ti,ab OR Belarus:ti,ab OR Belorussian:ti,ab OR Belorussia:ti,ab OR Belize:ti,ab OR Bhutan:ti,ab OR Bolivia:ti,ab OR Bosnia:ti,ab OR Herzegovina:ti,ab OR Hercegovina:ti,ab OR Botswana:ti,ab OR Brasil:ti,ab OR Brazil:ti,ab OR Bulgaria:ti,ab OR 'Burkina Faso':ti,ab OR 'Burkina Fasso':ti,ab OR 'Upper Volta':ti,ab OR Burundi:ti,ab OR Urundi:ti,ab OR Cambodia:ti,ab OR 'Khmer Republic':ti,ab OR Kampuchea:ti,ab OR Cameroon:ti,ab OR Cameroons:ti,ab OR Cameron:ti,ab OR Camerons:ti,ab OR 'Cape Verde':ti,ab OR 'Central African Republic':ti,ab OR Chad:ti,ab OR Chile:ti,ab OR China:ti,ab OR Colombia:ti,ab OR Comoros:ti,ab OR 'Comoro Islands':ti,ab OR Comores:ti,ab OR Mayotte:ti,ab OR Congo:ti,ab OR Zaire:ti,ab OR 'Costa Rica':ti,ab OR 'Cote d`Ivoire' OR 'Ivory Coast':ti,ab OR Croatia:ti,ab OR Cuba:ti,ab OR Cyprus:ti,ab OR Czechoslovakia:ti,ab OR 'Czech Republic':ti,ab OR Slovakia:ti,ab OR 'Slovak Republic':ti,ab OR Djibouti:ti,ab OR 'French Somaliland':ti,ab OR Dominica:ti,ab OR 'Dominican Republic':ti,ab OR 'East Timor':ti,ab OR 'East Timur':ti,ab OR 'Timor Leste':ti,ab OR Ecuador:ti,ab OR Egypt:ti,ab OR 'United Arab Republic':ti,ab OR El Salvador:ti,ab OR Eritrea:ti,ab OR Estonia:ti,ab OR Ethiopia:ti,ab OR Fiji:ti,ab OR Gabon:ti,ab OR 'Gabonese Republic':ti,ab OR Gambia:ti,ab OR Gaza:ti,ab OR 'Georgia Republic':ti,ab OR 'Georgian Republic':ti,ab OR Ghana:ti,ab OR 'Gold Coast':ti,ab OR Greece:ti,ab OR Grenada:ti,ab OR Guatemala:ti,ab OR Guinea:ti,ab OR Guam:ti,ab OR Guiana:ti,ab OR Guyana:ti,ab OR Haiti:ti,ab OR Honduras:ti,ab OR Hungary:ti,ab OR India:ti,ab OR Maldives:ti,ab OR Indonesia:ti,ab OR Iran:ti,ab OR Iraq:ti,ab OR 'Isle of Man':ti,ab OR Jamaica:ti,ab OR Jordan:ti,ab OR Kazakhstan:ti,ab OR Kazakh:ti,ab OR Kenya:ti,ab OR Kiribati:ti,ab OR Korea:ti,ab OR Kosovo:ti,ab OR Kyrgyzstan:ti,ab OR Kirghizia:ti,ab OR 'Kyrgyz Republic':ti,ab OR Kirghiz:ti,ab OR Kirgizstan:ti,ab OR 'Lao PDR':ti,ab OR Laos:ti,ab OR Latvia:ti,ab OR Lebanon:ti,ab OR Lesotho:ti,ab OR Basutoland:ti,ab OR Liberia:ti,ab OR Libya:ti,ab OR Lithuania:ti,ab OR Macedonia:ti,ab OR Madagascar:ti,ab OR 'Malagasy Republic':ti,ab OR Malaysia:ti,ab OR Malaya:ti,ab OR Malay:ti,ab OR Sabah:ti,ab OR Sarawak:ti,ab OR Malawi:ti,ab OR Nyasaland:ti,ab OR Mali:ti,ab OR Malta:ti,ab OR 'Marshall Islands':ti,ab OR Mauritania:ti,ab OR Mauritius:ti,ab OR melanesia:ab,ti OR 'Agalega Islands':ti,ab OR Mexico:ti,ab OR Micronesia:ti,ab OR 'Middle East':ti,ab OR Moldova:ti,ab OR Moldovia:ti,ab OR Moldovian:ti,ab OR Mongolia:ti,ab OR Montenegro:ti,ab OR Morocco:ti,ab OR Ifni:ti,ab OR Mozambique:ti,ab OR Myanmar:ti,ab OR Myanma:ti,ab OR Burma:ti,ab OR Namibia:ti,ab OR Nepal:ti,ab OR 'Netherlands Antilles':ti,ab OR 'New Caledonia':ti,ab OR Nicaragua:ti,ab OR Niger:ti,ab OR Nigeria:ti,ab OR 'Northern Mariana Islands':ti,ab OR Oman:ti,ab OR Muscat:ti,ab OR Pakistan:ti,ab OR Palau:ti,ab OR Palestine:ti,ab OR Panama:ti,ab OR Paraguay:ti,ab OR Peru:ti,ab OR Philippines:ti,ab OR Philipines:ti,ab OR Phillipines:ti,ab OR Phillippines:ti,ab OR Poland:ti,ab OR Portugal:ti,ab OR 'Puerto Rico':ti,ab OR Romania:ti,ab OR Rumania:ti,ab OR Roumania:ti,ab OR Russia:ti,ab OR Russian:ti,ab OR Rwanda:ti,ab OR Ruanda:ti,ab OR 'Saint Kitts':ti,ab OR 'St Kitts':ti,ab OR Nevis:ti,ab OR 'Saint Lucia':ti,ab OR 'St Lucia':ti,ab OR 'Saint Vincent':ti,ab OR 'St Vincent':ti,ab OR Grenadines:ti,ab OR Samoa:ti,ab OR 'Samoan Islands':ti,ab OR 'Navigator Island':ti,ab OR 'Navigator Islands':ti,ab OR 'Sao Tome':ti,ab OR 'Saudi Arabia':ti,ab OR Senegal:ti,ab OR Serbia:ti,ab OR Montenegro:ti,ab OR Seychelles:ti,ab OR 'Sierra Leone':ti,ab OR Slovenia:ti,ab OR 'Sri Lanka':ti,ab OR Ceylon:ti,ab OR 'Solomon Islands':ti,ab OR Somalia:ti,ab OR Sudan:ti,ab OR Suriname:ti,ab OR Surinam:ti,ab OR Swaziland:ti,ab OR Syria:ti,ab OR Syrian:ti,ab OR Tajikistan:ti,ab OR Tadzhikistan:ti,ab OR Tadjikistan:ti,ab OR Tadzhik:ti,ab OR Tanzania:ti,ab OR Thailand:ti,ab OR Togo:ti,ab OR 'Togolese Republic':ti,ab OR Tonga:ti,ab OR Trinidad:ti,ab OR Tobago:ti,ab OR Tunisia:ti,ab OR Turkey:ti,ab OR Turkmenistan:ti,ab OR Turkmen:ti,ab OR Tuvalu:ti,ab OR Uganda:ti,ab OR Ukraine:ti,ab OR Uruguay:ti,ab OR USSR:ti,ab OR 'Soviet Union':ti,ab OR 'Union of Soviet Socialist Republics':ti,ab OR Uzbekistan:ti,ab OR Uzbek OR Vanuatu:ti,ab OR 'New Hebrides':ti,ab OR Venezuela:ti,ab OR Vietnam:ti,ab OR 'Viet Nam':ti,ab OR 'West Bank':ti,ab OR Yemen:ti,ab OR Yugoslavia:ti,ab OR Zambia:ti,ab OR Zimbabwe:ti,ab OR Rhodesia:ti,ab OR 'developing country'/exp OR 'Africa'/de OR 'Africa south of the Sahara'/de OR 'North Africa'/de OR 'Central Africa'/de OR 'Asia'/de OR 'South Asia'/de OR 'Southeast Asia'/de OR 'South America'/de OR 'Central America'/de OR 'South and Central America'/de OR 'Atlantic islands'/de OR 'Caribbean Islands'/de OR 'Pacific islands'/de OR 'Indian Ocean'/de OR 'Eastern Europe'/de OR Afghanistan/exp OR Albania/exp OR Algeria/exp OR 'American Samoa'/exp OR Angola/exp OR 'Antigua and Barbuda'/exp OR Argentina/exp OR Armenia/exp OR Azerbaijan/exp OR Bahrain/exp OR Bangladesh/exp OR Barbados/exp OR Benin/exp OR 'Belarus'/exp OR 'Baltic States'/exp OR Belize/exp OR Bhutan/exp OR Bolivia/exp OR 'Bosnia and Herzegovina'/exp OR Botswana/exp OR Brazil/exp OR Bulgaria/exp OR 'Burkina Faso'/exp OR Burundi/exp OR Cambodia/exp OR Cameroon/exp OR 'Cape Verde'/exp OR 'Central African Republic'/exp OR Chad/exp OR Chile/exp OR China/exp OR Colombia/exp OR Comoros/exp OR Congo/exp OR 'Costa Rica'/exp OR 'Cote d`Ivoire'/exp OR Croatia/exp OR Cuba/exp OR Cyprus/exp OR Czechoslovakia/exp OR 'Czech Republic'/exp OR Slovakia/exp OR Djibouti/exp OR 'Democratic Republic Congo'/exp OR Dominica/exp OR 'Dominican Republic'/exp OR 'Timor-Leste'/exp OR Ecuador/exp OR Egypt/exp OR 'El Salvador'/exp OR Eritrea/exp OR Estonia/exp OR Ethiopia/exp OR 'French Guiana'/exp OR Fiji/exp OR Gabon/exp OR Gambia/exp OR 'Georgia (Republic) '/exp OR Ghana/exp OR Greece/exp OR Grenada/exp OR Guatemala/exp OR Guinea/exp OR Guinea-Bissau/exp OR Guam/exp OR Guyana/exp OR Haiti/exp OR Honduras/exp OR Hungary/exp OR India/exp OR Indonesia/exp OR Iran/exp OR Iraq/exp OR Jamaica/exp OR Jordan/exp OR Kazakhstan/exp OR Kenya/exp OR Korea/exp OR Kyrgyzstan/exp OR Laos/exp OR Latvia/exp OR Lebanon/exp OR Lesotho/exp OR Liberia/exp OR 'Libyan Arab Jamahiriya'/exp OR Lithuania/exp OR 'Macedonia (republic)'/exp OR Madagascar/exp OR Malaysia/exp OR Malawi/exp OR Mali/exp OR Malta/exp OR Mauritania/exp OR Mauritius/exp OR "Melanesia"/exp OR Mexico/exp OR 'Federated States of Micronesia'/exp OR 'Middle East'/de OR Moldova/exp OR Mongolia/exp OR Montenegro/exp OR Morocco/exp OR Mozambique/exp OR Myanmar/exp OR Namibia/exp OR Nepal/exp OR 'Netherlands Antilles'/exp OR 'New Caledonia'/exp OR Nicaragua/exp OR Niger/exp OR Nigeria/exp OR 'North Korea'/exp OR Oman/exp OR Pakistan/exp OR Palau/exp OR Panama/exp OR 'Papua New Guinea'/exp OR Paraguay/exp OR Peru/exp OR Philippines/exp OR Poland/exp OR Portugal/exp OR 'Puerto Rico'/exp OR Romania/exp OR 'Russian Federation'/exp OR Rwanda/exp OR 'Saint Kitts and Nevis'/exp OR 'Saint Lucia'/exp OR 'Saint Vincent and the Grenadines'/exp OR 'Samoan Islands'/exp OR Samoa/exp OR 'Saudi Arabia'/exp OR Senegal/exp OR Serbia/exp OR 'Montenegro (republic)'/exp OR Seychelles/exp OR 'Sierra Leone'/exp OR Slovenia/exp OR 'Sri Lanka'/exp OR Somalia/exp OR 'South Korea'/exp OR 'South Africa'/exp OR Sudan/exp OR Suriname/exp OR Swaziland/exp OR 'Syrian Arab Republic'/exp OR Tajikistan/exp OR Tanzania/exp OR Thailand/exp OR Togo/exp OR Tonga/exp OR 'Trinidad and Tobago'/exp OR Tunisia/exp OR 'Turkey (republic)'/exp OR Turkmenistan/exp OR Uganda/exp OR Ukraine/exp OR Uruguay/exp OR USSR/exp OR Uzbekistan/exp OR Vanuatu/exp OR Venezuela/exp OR 'Viet Nam'/exp OR Yemen/exp OR Yugoslavia/exp OR 'Yugoslavia (pre-1992)'/exp OR Zambia/exp OR Zimbabwe/exp

**Concept 5: Animals**

("animals"[MeSH Terms] NOT ("humans"[MeSH Terms] AND "animals"[MeSH Terms]))
