# Supplementary material for: Cost of Nine Pediatric Infectious Illnesses in Low- and Middle-Income Countries: A Systematic Review of Cost-of-Illness Studies
Source: Pharmacoeconomics. 2020 Aug 4;38(10):1071–94. doi: 10.1007/s40273-020-00940-4 (PMC7578143; doi:10.1007/s40273-020-00940-4)
Supplement: Supplementary file 4 — (PDF 104 kb) [file 40273_2020_940_MOESM4_ESM.pdf]

Appendix IV

TABLE IV.1: Reported costs by perspective

Perspective	Direct costs		Indirect costs
	Medical	Non-medical	
**Household**	Consultation or registration fees<br>Cost of investigations/diagnosis (radiology, laboratory tests)<br>Cost of hospitalization/bed for the patient<br>Cost of medications<br>Cost of medical supplies (oxygen tanks, nebulizer)<br>Cost of traditional healing and non-institutionalized healthcare<br>Other medical costs [a]	Cost of transportation to and from the healthcare facilities for the patient and the caregiver<br>Cost of transportation for additional visitors to the patient during hospitalization<br>Cost of meals for the patient and the caregiver during hospitalization<br>Cost of lodging for the caregiver while the patient is hospitalized<br>Cost of childcare for the household while the caregiver is away to the healthcare facility caring for the patient<br>Cost of household supplies (diapers, soap)<br>Miscellaneous costs (telephone calls, gifts from visitors)	Productivity loss for the caregiver related to the acute episode of illness<br>Productivity loss for the caregiver related to disability due to the illness
	Service costs		
**Government & healthcare facilities**	Cost of investigations/diagnosis (radiology, laboratory tests)<br>Cost of hospitalization/bed<br>Cost of medications<br>Cost of personnel (full-time, part-time and on-call staff)<br>Cost of medical supplies<br>Cost of overhead (building maintenance, utilities)<br>Capital costs (infrastructure, furniture, vehicles, laboratory and radiology instruments)		
**Health insurance**	Amount paid by health insurance		

Notes: [a] As reported in the articles.
